# Reported clinical breast examination activity in Bangladesh, 2019–2025: Temporal trends, geographic variation, positivity, and reporting completeness

**DOI:** 10.1371/journal.pone.0361910

**Published:** 2026-10-29

**Authors:** Sourov Das, Simran Patra, Shubham Om, Sushmita Kundu

**Affiliations:** 1 Comilla Medical College and Hospital, Cumilla, Bangladesh; 2 Far Eastern Federal University, School of Medicine, Vladivostok, Russian Federation; 3 Tbilisi State Medical University, Tbilisi, Georgia; Tangshan People's Hospital, CHINA

## Abstract

**Background:**

Routine clinical breast examination (CBE) services contribute to breast cancer early-detection activities in Bangladesh, but national program performance and reporting completeness are poorly characterized. We assessed reported CBE activity, geographic variation, positivity, reporting completeness, and temporal trends from 2019 through 2025.

**Methods:**

We retrospectively analyzed routine CBE program data from 1,209 location-specific eligible facilities across Bangladesh from January 2019 through December 2025. Because individual-level identifiers were unavailable, records were analyzed as CBE events rather than unique women screened. Reported CBE examination rates used estimated populations of women aged 30–69 years, with district denominators proportionally allocated using census-based population distributions. Reporting completeness was defined as submitted facility-month reports divided by expected reports. Interrupted time-series regression assessed changes associated with March 2020 using calendar-month fixed effects and heteroskedasticity- and autocorrelation-consistent standard errors.

**Results:**

A total of 3,193,080 reported CBE events were recorded. The reported examination rate increased from 0.3% in 2019 to 2.4% in 2023, then declined to 1.8% in 2024 and 1.1% in 2025. In 2025, division-level rates ranged from 0.7% to 1.7% and district-level rates from 0.2% to 2.8%. Overall positivity was 1.0% (31,624 positive events), reaching 1.6% in 2025. Mean annual reporting completeness rose from 12.1% in 2019 to 42.2% in 2024 and was 39.7% in 2025; no month reached 60% national completeness. Interrupted time-series analysis found no significant immediate level change (14,416 events; 95% CI −7,438–36,269; P = 0.196) or post-intervention slope change (−375 events/month; 95% CI −1,673–923; P = 0.571).

**Conclusions:**

Reported CBE activity expanded through 2023 but subsequently declined, with marked geographic variation and persistently incomplete reporting. These findings describe reported program activity rather than true population screening coverage. Strengthening routine reporting, individual-level tracking, quality assurance, and referral follow-up is needed for more reliable program evaluation.

## Introduction

Breast cancer is the most commonly diagnosed cancer among women worldwide and contributes substantially to cancer morbidity and mortality, particularly in low- and middle-income countries (LMICs) [1–3]. Across South Asia, breast cancer incidence and mortality have increased, while delayed presentation and barriers to timely diagnosis and treatment remain common [4–8]. In higher-income settings, reductions in breast cancer mortality have reflected a combination of earlier diagnosis, organized screening in eligible populations, and access to effective treatment [9].

In Bangladesh, breast cancer is the most frequently reported malignancy among women and accounts for a substantial proportion of cancers recorded at the National Institute of Cancer Research and Hospital (NICRH) [10]. Early detection remains challenging because of limited awareness, financial barriers, social stigma, delayed care seeking, uneven diagnostic capacity, and shortages of trained personnel [7,11–13]. Population-based mammographic screening requires substantial infrastructure and diagnostic and treatment capacity and is not universally feasible in resource-constrained settings [14]. The Bangladesh National Cancer Control Strategy and Plan of Action 2009–2015 considered population mammographic screening impractical in the national context at that time and proposed clinical breast examination (CBE) as a feasible early-detection approach [15].

Bangladesh subsequently incorporated CBE into public-sector breast cancer early-detection activities. Government-supported services have been implemented through district hospitals and progressively expanded toward upazila health complexes, which are subdistrict-level public health facilities. By 2020, the government, with support from the World Health Organization (WHO), reported CBE services across all districts and expansion to hundreds of upazila health complexes [16]. This service model is also relevant to the WHO Global Breast Cancer Initiative framework, which emphasizes early detection, timely diagnosis, and comprehensive breast cancer management [17]. The public program evaluated in the present study therefore reflects CBE-based service delivery rather than a population mammography program. Women with abnormal examinations require further diagnostic evaluation and referral, including imaging and tissue diagnosis where indicated. Higher-level cancer facilities provide such services; NICRH, for example, includes a dedicated breast surgical unit and diagnostic and oncologic services [18]. However, routine national CBE data do not contain individual-level information on referral completion, diagnostic confirmation, treatment, or survival.

CBE has been investigated as a pragmatic early-detection strategy in settings where organized mammography is difficult to implement [19–28]. Nevertheless, interpretation of routine CBE program data requires caution. Counts reported by health facilities may represent examination events rather than unique individuals, incomplete facility reporting can substantially affect apparent population rates, and facility location does not necessarily indicate participants’ residence. Consequently, routine program data are better suited to describing reported service activity and geographic variation than estimating true population screening coverage.

Routine digital reporting from Bangladesh’s CBE program has been available nationally from 2019 onward, yet longitudinal patterns in reported activity, reporting completeness, geographic variation, and CBE positivity have not been comprehensively characterized. We therefore analyzed national routine program data from January 2019 through December 2025. Our objectives were to describe temporal and geographic variation in reported CBE activity, quantify positivity and reporting completeness, examine the distribution of activity across facility types, and assess whether monthly reported activity changed following March 2020 using interrupted time-series analysis.

## Methods

### Study design and setting

We conducted a retrospective analysis of routinely collected aggregate clinical breast examination (CBE) program data from Bangladesh from January 2019 through December 2025. The national program included a network of 1,209 location-specific eligible reporting facilities comprising medical college hospitals, university hospitals, district hospitals, upazila health complexes, and other government-affiliated facilities. An upazila is a subdistrict administrative unit in Bangladesh, and an upazila health complex is a public-sector health facility serving that level of the health system.

The available data were aggregated at the facility-month level and did not contain individual-level identifiers. Consequently, repeat examinations of the same individual could not be identified, and the analytic numerator was interpreted as reported CBE examination events rather than unique women screened. Participant place of residence was also unavailable; geographic analyses therefore describe the location of reporting facilities and administrative areas rather than individual residence.

### Data sources and reporting structure

The primary data were obtained from the national cervical and breast cancer early-detection program database maintained by the Directorate General of Health Services [29]. Participating facilities reported routine program data electronically on a monthly basis to the national program database. Routine program information was consolidated as monthly facility-level records. Available fields included reporting period, division, district, upazila, facility, facility category, examination type, number of positive examinations, number of negative examinations, and total examinations reported.

The original program dataset through September 2025 was accessed for research purposes on December 5, 2025. Updated program exports covering October through December 2025 were accessed on September 1, 2026 and incorporated into the revised analysis. Records were restricted to CBE examinations, administrative and facility-name variants were harmonized, and facility categories were standardized. For reporting-completeness calculations, facility identity was defined by district and normalized facility name so that same-named facilities in different districts were counted separately. We checked for duplicate facility-month records, missing required variables, and arithmetic inconsistencies between positive, negative, and total examination counts. No duplicate facility-month records or arithmetic inconsistencies remained in the final analytic dataset, and no required analytic fields were missing. Missing facility-month reports were retained as missing reports and were not imputed.

Seven facilities first appeared in the October–December 2025 exports. The primary reporting-completeness analysis used the 1,209 location-specific facilities present in the baseline roster; a sensitivity analysis allowing 1,216 eligible facilities for October–December 2025 produced a negligible change in the 2025 completeness estimate.

For contextualization, summary indicators were obtained from the National Institute of Cancer Research and Hospital (NICRH) Hospital-Based Cancer Registry for 2018–2020 [10]. These registry data were analyzed descriptively and were not linked at the individual level to CBE program records. They therefore could not be used to estimate diagnostic confirmation, treatment completion, stage, or survival among individuals undergoing CBE.

### Population denominators

Annual national denominators were based on age- and sex-specific population estimates from the United Nations World Population Prospects 2022 Revision for women aged 30–69 years [30]. District-level eligible populations were estimated by proportionally allocating national eligible-population totals according to female population distributions from the Bangladesh Bureau of Statistics 2022 Population and Housing Census [31]. Division-level denominators were obtained by aggregating corresponding district estimates.

Because district-specific age distributions for women aged 30–69 years were unavailable in the source data, this proportional allocation assumes that the age distribution of women is similar across districts. We therefore treated district-level rates as approximate measures and examined women aged 30–64 years as an alternative denominator in sensitivity analysis.

### Outcomes

The primary outcome was the reported CBE examination rate, calculated as the number of reported CBE examination events divided by the estimated population of women aged 30–69 years for the corresponding year and geographic area. Because facility reporting was incomplete and individual-level deduplication was unavailable, this measure was not interpreted as true population screening coverage.

Secondary outcomes were CBE positivity, reporting completeness, geographic variation, and distribution of activity by facility type. Positivity was calculated as positive CBE events divided by all reported CBE events. A positive CBE represented an abnormal examination recorded by the program and did not indicate a confirmed breast cancer diagnosis.

Reporting completeness was defined as the number of submitted facility-month reports divided by the number of expected facility-month reports from the 1,209 location-specific eligible facilities. Completeness was summarized nationally by month and year and geographically for 2025.

Facility categories were medical college hospital, university hospital, upazila health complex, district hospital, and other. Facility type was used only to describe where reported CBE activity occurred and was not used as a proxy for participant residence.

### Statistical analysis

National reported CBE events and examination rates were summarized annually from 2019 through 2025. Geographic variation was assessed across divisions and districts, and CBE activity was summarized by facility category. Positivity was calculated annually and monthly.

Sensitivity analyses included use of women aged 30–64 years as the eligible denominator and exclusion of the 60 highest-volume facilities, corresponding to approximately 5% of the 1,209-facility network. To evaluate whether temporal patterns were driven by changes in the set of reporting facilities, we additionally analyzed fixed cohorts of facilities reporting in at least 9 months of every year from 2019 through 2025 and, in a secondary sensitivity analysis, facilities reporting in at least 6 months of every year. As an exploratory analysis, Spearman rank correlation was used to assess the association between district-level reporting completeness and reported CBE examination rates in 2025.

Interrupted time-series analysis was performed using monthly national CBE event counts from January 2019 through December 2025. March 2020 was specified as the interruption point. The segmented regression included continuous time, an indicator for the post-March 2020 period, time after the interruption, and calendar-month fixed effects to account for seasonality. Newey–West heteroskedasticity- and autocorrelation-consistent standard errors with 12 monthly lags were used to account for serial correlation. The principal parameters of interest were the immediate level change and change in monthly slope after March 2020.

Sensitivity analyses repeated the interrupted time-series model using CBE events per reporting facility and the fixed cohort of 48 facilities that reported during at least 9 months of every study year. Additional robustness checks used April 2020 as an alternative interruption point and an autoregressive error specification. Statistical tests were two-sided, with P < 0.05 considered statistically significant. Analyses were conducted in Python.

Reporting of this observational study followed the STROBE recommendations, and a completed STROBE checklist is provided as Supporting Information.

### Ethics statement

This study involved secondary analysis of routinely collected, de-identified aggregate program data combined with publicly available population statistics. The investigators did not have access to individual-level identifiers during or after data collection. As no individual-level identifiable participant data were included in the analytic dataset, institutional review board approval was not required.

## Results

### National reported CBE activity

From January 2019 through December 2025, the final analytic dataset contained 32,178 facility-month records and 3,193,080 reported CBE examination events. Annual reported activity increased from 114,060 events in 2019 to a peak of 844,592 in 2023, before declining to 630,514 in 2024 and 417,288 in 2025.

The corresponding reported CBE examination rate increased from 0.3% in 2019 to 0.5% in 2020, 1.3% in 2021, 1.8% in 2022, and a peak of 2.4% in 2023, before declining to 1.8% in 2024 and 1.1% in 2025 (Table 1; Fig 1). The mean annual reported examination rate over the seven-year period was approximately 1.3%. These rates represent reported examination activity rather than confirmed population screening coverage.

**Table 1:** National reported clinical breast examination activity, Bangladesh 2019–2025.

Year	Reported CBE events (n)	Eligible women aged 30–69 years (N)	Reported CBE examination rate (%)
2019	114,060	32,907,395	0.3
2020	164,638	33,050,942	0.5
2021	430,924	33,158,954	1.3
2022	591,064	33,655,903	1.8
2023	844,592	34,585,364	2.4
2024	630,514	35,574,839	1.8
2025	**417,288**	**36,612,684**	**1.1**

CBE, clinical breast examination. The numerator represents reported CBE examination events rather than confirmed unique women because individual-level identifiers were unavailable and repeat examinations could not be identified. The reported CBE examination rate was calculated as reported CBE events divided by the estimated population of women aged 30–69 years. Because facility reporting was incomplete, these values should not be interpreted as true population screening coverage.

**Fig 1:**
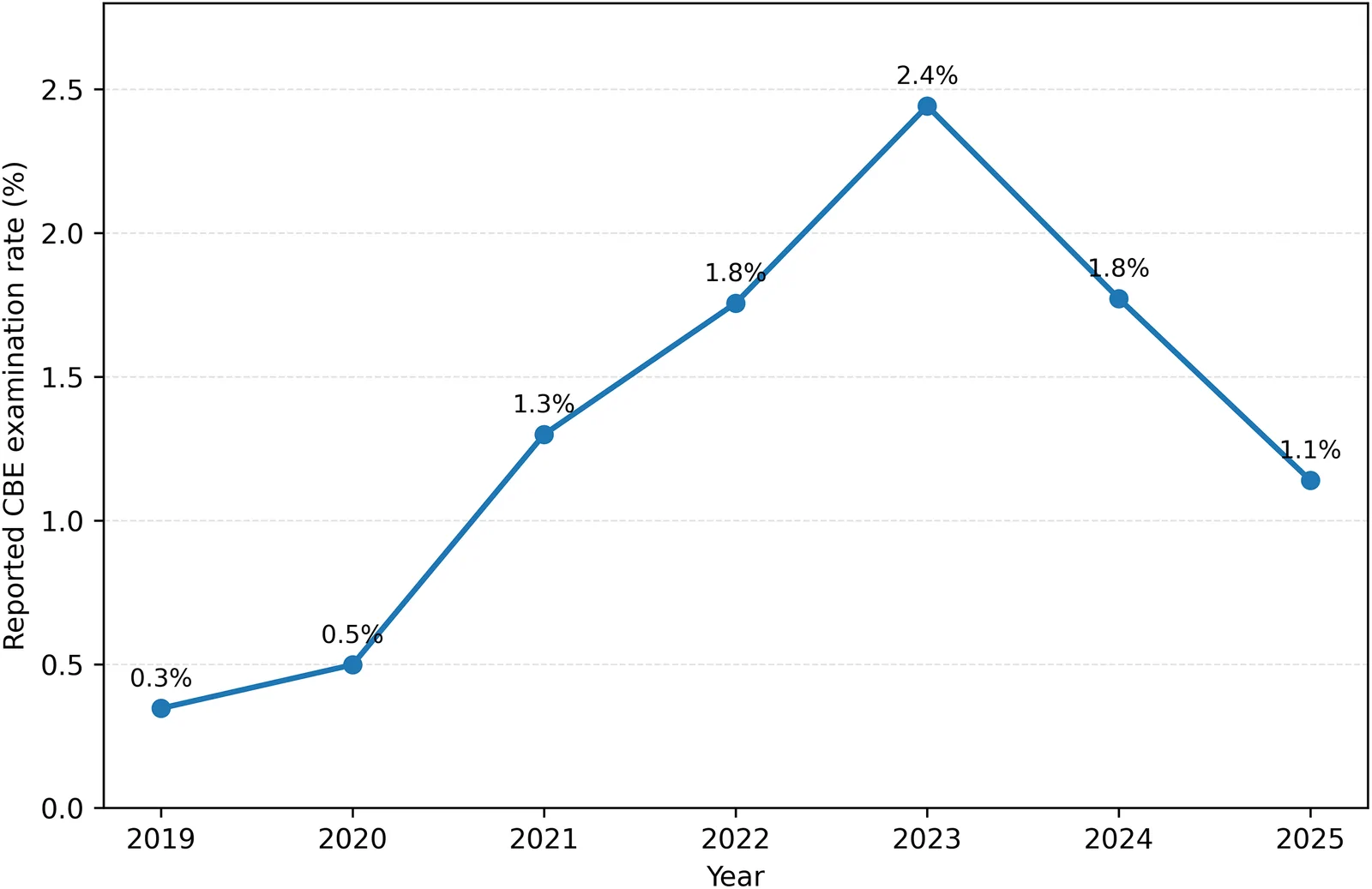
Annual reported clinical breast examination activity in Bangladesh, 2019–2025. Reported CBE examination rates were calculated as the number of reported CBE examination events divided by the estimated population of women aged 30–69 years. The 2025 estimate represents the complete January–December calendar year. Because facility reporting was incomplete and individual-level identifiers were unavailable, these rates should not be interpreted as true population screening coverage.

### Geographic variation

Substantial geographic variation was observed. At the division level, reported CBE examination rates in 2019 ranged from <0.1% in Sylhet to 0.5% in Dhaka. In 2023, rates ranged from 1.6% in Mymensingh to 3.1% in Khulna. In the complete 2025 calendar year, rates ranged from 0.7% in Mymensingh (18,183 events among an estimated 2,738,471 eligible women) to 1.7% in Khulna (65,684 of 3,859,986) (Table 2; Fig 2).

**Table 2:** Division-level extremes in reported clinical breast examination activity, Bangladesh, 2019, 2023, and 2025.

Year	Lowest-rate division	Reported CBE events (n)	Eligible women (N)	Reported CBE examination rate (%)	Highest-rate division	Reported CBE events (n)	Eligible women (N)	Reported CBE examination rate (%)
2019	Sylhet	1,206	2,229,874	<0.1	Dhaka	45,340	8,576,309	0.5
2023	Mymensingh	40,118	2,586,836	1.6	Khulna	114,079	3,646,250	3.1
2025	**Mymensingh**	**18,183**	**2,738,471**	**0.7**	**Khulna**	**65,684**	**3,859,986**	**1.7**

CBE, clinical breast examination. Rates represent reported CBE examination events divided by the estimated population of women aged 30–69 years within each division. Because facility reporting was incomplete and individual-level identifiers were unavailable, these values should not be interpreted as true population screening coverage.

**Fig 2:**
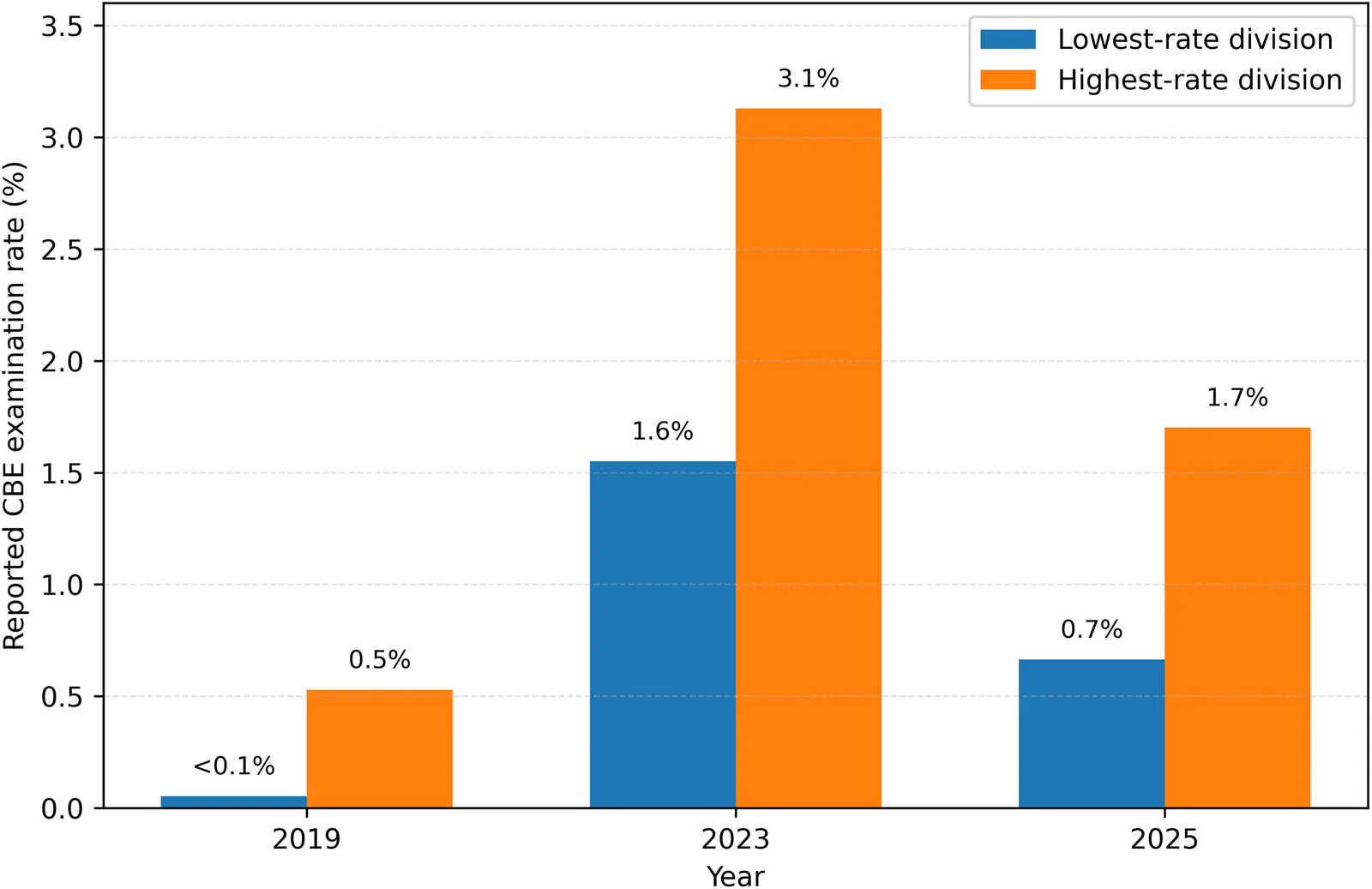
Lowest and highest division-level reported clinical breast examination activity in Bangladesh, 2019, 2023, and 2025. Bars show the lowest and highest division-level reported CBE examination rates for each selected year. Reported CBE examination rates were calculated as the number of reported CBE examination events divided by the estimated population of women aged 30–69 years within each division. The 2025 estimates represent the complete January–December calendar year. Because facility reporting was incomplete and individual-level identifiers were unavailable, these rates should not be interpreted as true population screening coverage.

Variation was greater at the district level. In 2019, reported examination rates ranged from <0.1% in Sunamganj to 1.2% in Joypurhat. In 2023, rates ranged from 0.8% in Sunamganj to 6.8% in Shariatpur. In 2025, the lowest rate was 0.2% in Kurigram (1,205 of 519,564) and the highest was 2.8% in Rajshahi (17,737 of 639,148) (Table 3; Fig 3). Full district- and division-level results are provided in the Supporting Information.

**Table 3:** District-level extremes in reported clinical breast examination activity, Bangladesh, 2019, 2023, and 2025.

Year	Lowest-rate district	Reported CBE events (n)	Eligible women (N)	Reported CBE examination rate (%)	Highest-rate district	Reported CBE events (n)	Eligible women (N)	Reported CBE examination rate (%)
2019	Sunamganj	1	541,531	<0.1	Joypurhat	2,199	190,892	1.2
2023	Sunamganj	4,481	569,144	0.8	Shariatpur	18,851	279,153	6.8
2025	**Kurigram**	**1,205**	**519,564**	**0.2**	**Rajshahi**	**17,737**	**639,148**	**2.8**

CBE, clinical breast examination. Rates represent reported CBE examination events divided by the estimated population of women aged 30–69 years within each district. District-level eligible populations were estimated using proportional allocation from national age-specific population estimates. Because facility reporting was incomplete and individual-level identifiers were unavailable, these values should not be interpreted as true population screening coverage.

**Fig 3:**
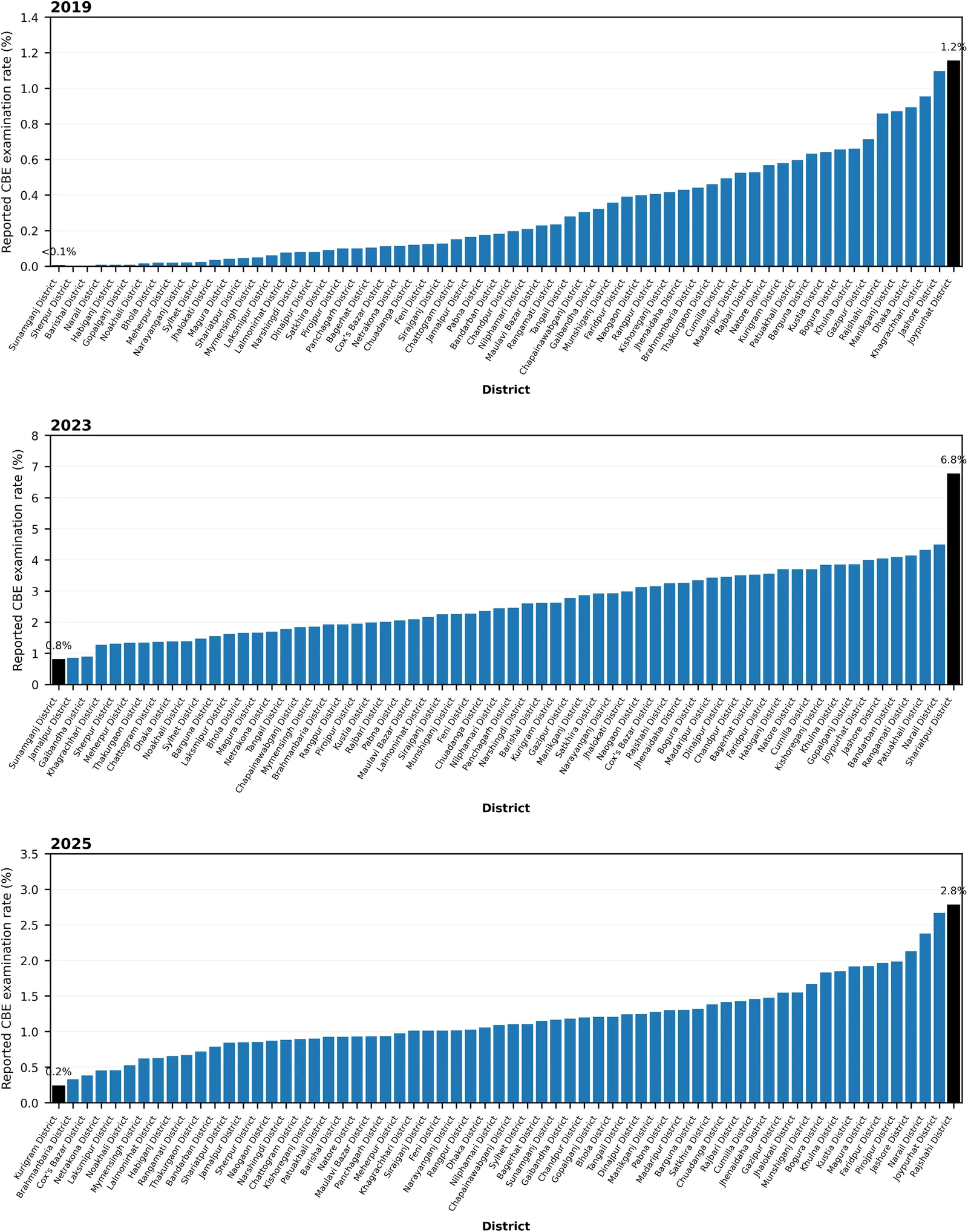
District-level distribution of reported clinical breast examination activity in Bangladesh, 2019, 2023, and 2025. Districts are ordered from the lowest to the highest reported CBE examination rate within each selected year. Reported CBE examination rates were calculated as the number of reported CBE examination events divided by the estimated population of women aged 30–69 years within each district. The 2025 panel represents the complete January–December calendar year. Because facility reporting was incomplete and individual-level identifiers were unavailable, these rates should not be interpreted as true population screening coverage.

### Distribution of activity by facility type

The distribution of reported CBE activity differed substantially by facility category (Table 4; Fig 4). Upazila health complexes accounted for the largest share of reported events in each selected year: 52.7% in 2019, 76.3% in 2023, and 58.3% in 2025. In 2025, district hospitals accounted for 21.2% of reported events, medical college hospitals for 15.3%, other facilities for 3.6%, and university hospitals for 1.6%.

**Table 4:** Distribution of reported clinical breast examination activity by facility type, Bangladesh, 2019, 2023, and 2025.

Facility type	2019, n (%)	2023, n (%)	2025, n (%)
Medical college hospitals	10,877 (9.5)	54,690 (6.5)	63,824 (15.3)
University hospitals	16,326 (14.3)	9,679 (1.1)	6,614 (1.6)
Upazila health complexes	60,140 (52.7)	644,115 (76.3)	243,307 (58.3)
District hospitals	22,657 (19.9)	119,375 (14.1)	88,557 (21.2)
Other facilities	4,060 (3.6)	16,733 (2.0)	14,986 (3.6)
**Total**	**114,060 (100)**	**844,592 (100)**	**417,288 (100)**

CBE, clinical breast examination. Percentages represent the proportion of all reported CBE examination events occurring within each facility category in the corresponding year. Facility type reflects the reporting facility and does not indicate participants’ place of residence.

**Fig 4:**
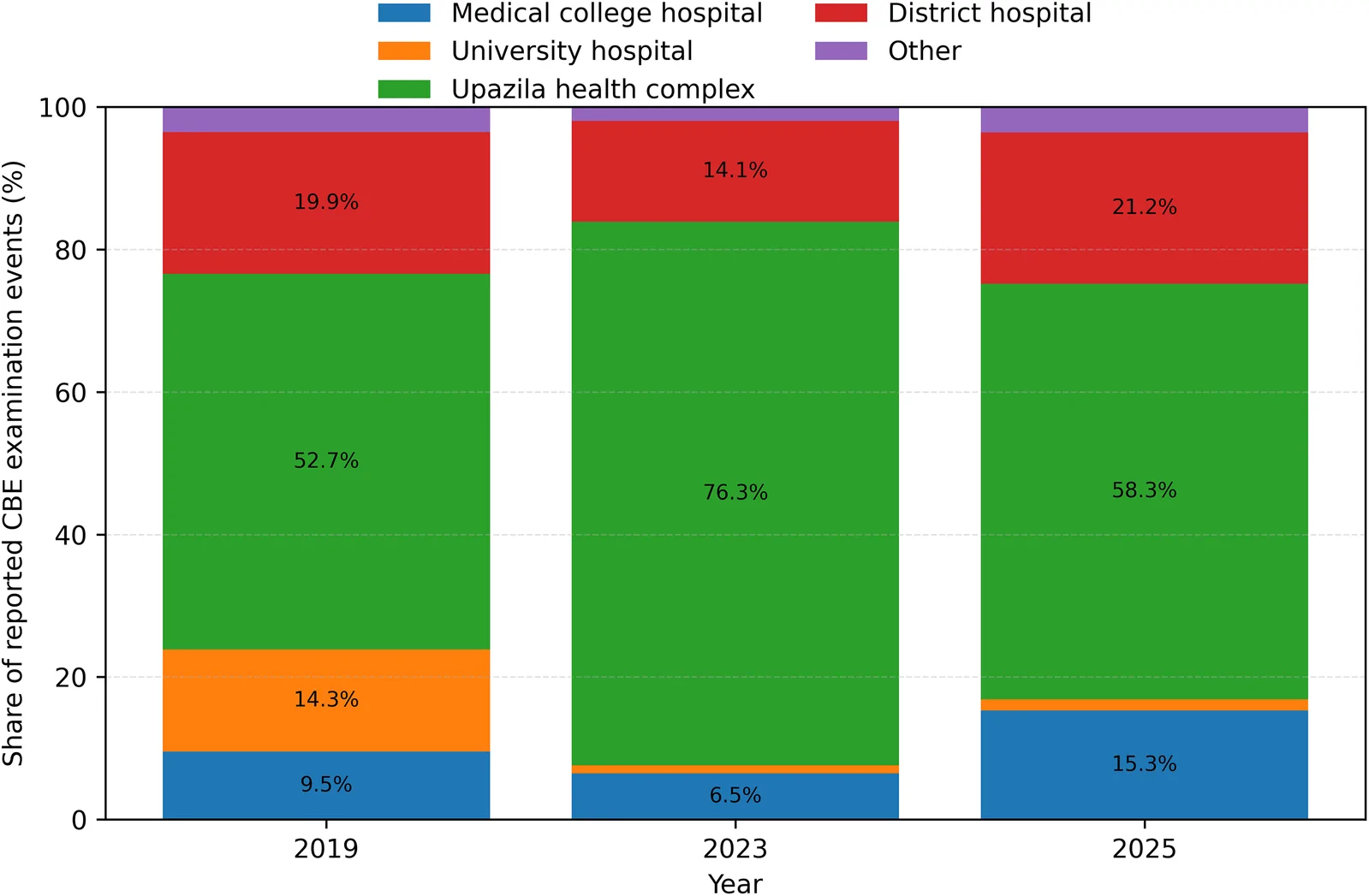
Distribution of reported clinical breast examination activity by facility type in Bangladesh, 2019, 2023, and 2025. Bars show the proportion of all reported CBE examination events occurring within each facility category in the corresponding year. Facility type refers to the reporting facility and does not indicate participants’ place of residence. The 2025 data represent the complete January–December calendar year.

These proportions describe the location and type of the reporting facility and do not represent the residence of individuals undergoing CBE.

### CBE positivity

Across 2019–2025, 31,624 positive CBE events were reported among 3,193,080 examinations, corresponding to an overall positivity of approximately 1.0%. Annual positivity was 1.1% in 2019, 0.8% in 2020, 0.6% in 2021, 0.8% in 2022, 0.9% in 2023, 1.2% in 2024, and 1.6% in 2025 (Table 5).

**Table 5:** Positivity of reported clinical breast examination events, Bangladesh, 2019–2025.

Year	Reported CBE events (N)	Positive CBE events (n)	Positivity (%)
2019	114,060	1,280	1.1
2020	164,638	1,382	0.8
2021	430,924	2,562	0.6
2022	591,064	4,518	0.8
2023	844,592	7,860	0.9
2024	630,514	7,519	1.2
2025	**417,288**	**6,503**	**1.6**

CBE, clinical breast examination. Positivity was calculated as positive CBE events divided by total reported CBE events. A positive CBE indicates a reported abnormal breast examination finding and does not represent a confirmed breast cancer diagnosis.

Monthly positivity ranged from approximately 0.4% to 2.5% across the study period (Fig 5). The highest monthly positivity occurred in May 2020, when the number of reported examinations was comparatively low. Positive CBE events represented abnormal examination findings and were not equivalent to confirmed breast cancer diagnoses.

**Fig 5:**
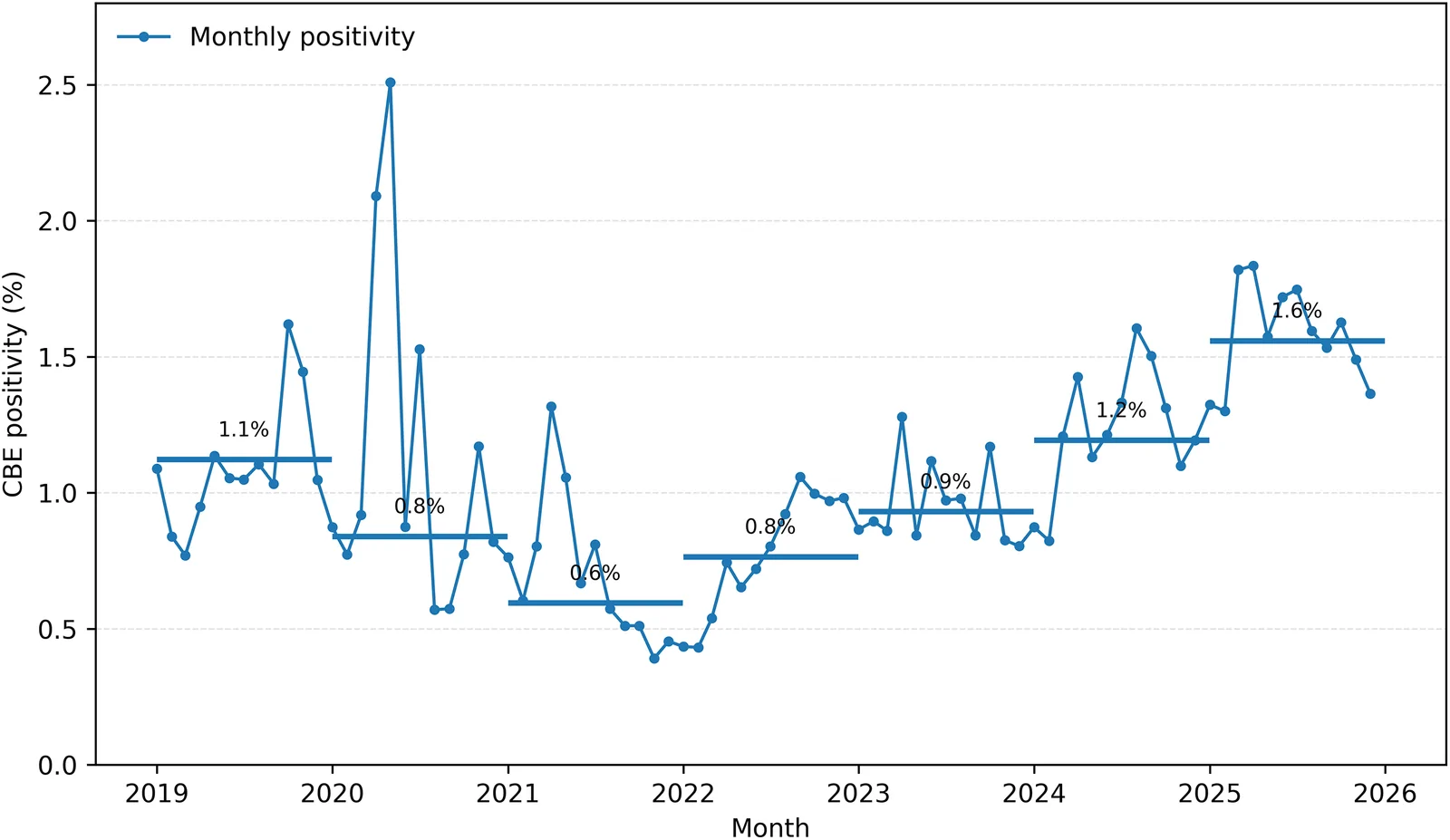
Monthly clinical breast examination positivity in Bangladesh, 2019–2025. The line shows monthly positivity, calculated as positive CBE events divided by total reported CBE examination events. Horizontal segments indicate the corresponding annual positivity for each calendar year. Monthly positivity ranged from approximately 0.4% to 2.5% during the study period. A positive CBE represents a reported abnormal breast examination finding and does not indicate a confirmed breast cancer diagnosis.

### Reporting completeness

Reporting remained incomplete throughout the study period. Mean annual completeness increased from 12.1% in 2019 to 17.6% in 2020, 31.1% in 2021, 37.0% in 2022, 42.0% in 2023, and 42.2% in 2024, before declining modestly to 39.7% in 2025 (Appendix Table A4). Across all 84 study months, no month reached the prespecified threshold of 60% national reporting completeness.

In 2025, mean reporting completeness varied across divisions from approximately 21.0% to 52.8% and across districts from approximately 9.4% to 100%. In an exploratory district-level analysis, reporting completeness was not significantly correlated with the reported CBE examination rate in 2025 (Spearman ρ = −0.151, P = 0.235).

### Sensitivity analyses

Sensitivity analyses demonstrated that several broad temporal findings persisted under alternative analytic assumptions (Appendix Tables A1 and A5). Using women aged 30–64 years as the denominator increased the mean annual reported examination rate from approximately 1.3% to 1.4%, without materially changing the temporal pattern.

Reported activity was concentrated among high-volume facilities. The 60 highest-volume facilities, representing approximately 5% of the 1,209-facility network, accounted for 30.3% of all reported CBE events. Excluding these facilities reduced the mean annual reported examination rate from approximately 1.3% to 0.9%, indicating meaningful concentration of program activity.

Among the 48 facilities reporting in at least 9 months of every study year, reported CBE events increased from 37,334 in 2019–121,515 in 2023 and then declined to 72,392 in 2025, a 40.4% decrease from 2023 to 2025. A broader fixed cohort of 118 facilities reporting in at least 6 months of every year showed a similar decline, from 260,265 events in 2023–157,461 in 2025, a 39.5% decrease. Between 2023 and 2025, submitted facility-month reports changed from 572 to 570 in the strict cohort and from 1,408–1,382 in the broader cohort, while CBE events per submitted report decreased from 212.4 to 127.0 and from 184.8 to 113.9, respectively (Appendix Table A5). Thus, the post-2023 decline was also observed within fixed sets of consistently reporting facilities.

### Interrupted time-series analysis

In the primary interrupted time-series model, the estimated immediate level change associated with March 2020 was + 14,416 events (95% CI −7,438–36,269; P = 0.196), while the post-intervention change in slope was −375 events per month (95% CI −1,673–923; P = 0.571) (Fig 6; Appendix Table A6). Neither parameter was statistically significant.

**Fig 6:**
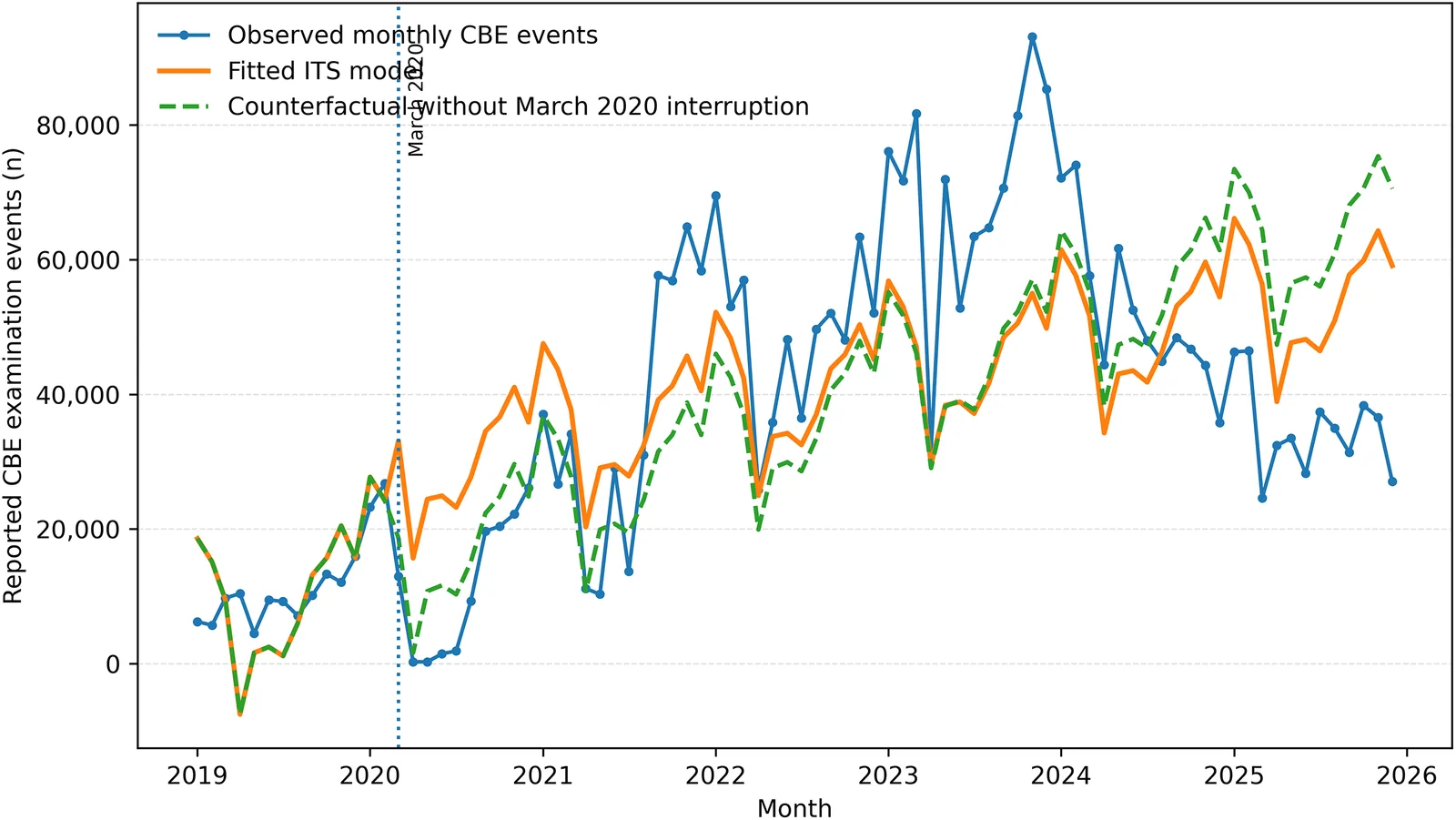
Interrupted time-series analysis of monthly reported clinical breast examination activity in Bangladesh, 2019–2025. The figure shows observed monthly reported CBE examination events, fitted values from the segmented regression model, and the counterfactual trajectory projected from the pre-intervention trend. March 2020 was specified as the intervention point. The model included calendar-month fixed effects to account for seasonality and Newey-West heteroskedasticity- and autocorrelation-consistent standard errors with 12 monthly lags. Neither the immediate level change nor the post-intervention slope change was statistically significant. Because the segmented linear model is unconstrained, fitted values may fall below zero in low-volume months; these values are model predictions rather than observed event counts.

Sensitivity analyses using events per reporting facility and the fixed cohort of 48 consistently reporting facilities likewise found no statistically significant immediate or slope change. Models using April 2020 as an alternative interruption point and an autoregressive error specification produced the same overall conclusion. Therefore, the interrupted time-series analysis did not provide statistically conclusive evidence of either an immediate or sustained change in reported CBE activity associated with the March 2020 interruption.

### Contextual NICRH registry indicators

Contextual data from the NICRH Hospital-Based Cancer Registry for 2018–2020 indicated that breast cancer accounted for approximately 13–14% of registered cancers, corresponding to roughly 1,500–1,640 cases annually (Table 6). More than three-quarters of patients were reported as untreated before presentation to NICRH. Among 1,590 registry cases with diagnostic information, 1,280 (80.5%) had histopathological confirmation, 243 (15.3%) had cytological confirmation, and 64 (4.0%) were diagnosed on radiological or clinical grounds alone.

**Table 6:** Contextual breast cancer registry indicators from NICRH, Bangladesh, 2018–2020.

Measure	n/N	%
Breast cancer cases among all registered cancers	~1,500–1,640 / ~ 12,000	13–14
Patients untreated before presentation to NICRH	~1,200 / ~ 1,600	>75
Histopathological confirmation	1,280 / 1,590	80.5
Cytological confirmation	243 / 1,590	15.3
Radiological or clinical diagnosis alone	64 / 1,590	4.0

NICRH, National Institute of Cancer Research and Hospital. These data are descriptive contextual indicators from the NICRH Hospital-Based Cancer Registry (2018–2020) and were not linked at the individual level to records from the national CBE program. They therefore should not be interpreted as outcomes of CBE screening or as nationally representative measures of treatment linkage.

These registry observations were not individually linked to the national CBE dataset and therefore cannot be interpreted as diagnostic, treatment, or survival outcomes among individuals represented in the CBE program.

## Discussion

In this analysis of routine program data from 1,209 location-specific eligible facilities across Bangladesh, more than 3.19 million CBE examination events were reported between 2019 and 2025. Reported activity increased substantially through 2023 and subsequently declined in 2024 and 2025. Geographic variation was considerable, activity was concentrated in a relatively small proportion of high-volume facilities, and upazila health complexes contributed the largest share of reported examinations in each selected year. Overall CBE positivity was approximately 1.0%. However, the central finding for interpretation is the persistent incompleteness of facility reporting. Consequently, the population rates reported in this study describe recorded program activity and should not be interpreted as estimates of true national screening coverage.

Reporting completeness improved markedly over time, from 12.1% in 2019 to approximately 42% in 2023–2024, but remained below 40% in 2025 and never reached 60% in any study month. This has important implications for both absolute rates and temporal comparisons. Missing facility reports cannot be assumed to represent zero CBE activity, and reporting may differ systematically by facility capacity, administrative performance, geography, and calendar period. We therefore did not impute unreported activity, because the available aggregate data did not provide a sufficiently defensible basis for assumptions about the missing observations. Instead, we explicitly present reporting completeness and interpret the estimates as reported activity.

The decrease after the 2023 peak nevertheless cannot be explained solely by changes in which facilities submitted reports. Among the fixed cohort of 48 facilities reporting during at least 9 months of every study year, reported examinations decreased by 40.4% between 2023 and 2025. A broader cohort of 118 consistently reporting facilities showed a similar 39.5% decrease. These analyses support the presence of a decline in recorded activity within consistently reporting facilities, although incomplete reporting prevents precise estimation of the magnitude of any population-level change. The concentration analysis also showed that approximately 5% of facilities accounted for 30.3% of all reported examinations. This suggests that national program activity depends disproportionately on a relatively small group of high-volume facilities and may therefore be sensitive to changes in performance at these sites.

Geographic variation should similarly be interpreted cautiously. Division- and district-level rates differed substantially, but these differences may reflect a combination of true service activity, reporting completeness, facility distribution, population-denominator assumptions, and local program implementation. District denominators were estimated by proportionally allocating the national population of women aged 30–69 years using census-based female population distributions because district-specific age distributions were unavailable. Furthermore, the program data identify the reporting facility rather than the residence of the person undergoing examination.

Upazila health complexes accounted for 76.3% of reported CBE events in 2023 and 58.3% in 2025, indicating that much of the recorded public-sector activity occurred at the subdistrict level. This pattern is consistent with the intended decentralization of CBE services within Bangladesh's public health system [15,16]. It should not, however, be interpreted as evidence that rural women had greater screening coverage than urban women. Individuals may seek care outside their area of residence, and services obtained through private or other facilities not represented in the program dataset would not be captured. Accordingly, patient-level residence and utilization data would be required for a valid assessment of rural-urban equity.

Overall CBE positivity was approximately 1.0%, lower than positivity reported in some CBE studies and early-detection programs in other LMIC settings [22,25–28,32–34]. This comparison must be made cautiously. The present dataset did not include participant age, breast cancer risk factors, symptoms, examiner training, quality-assurance measures, diagnostic confirmation, or referral completion. Differences in any of these factors could affect positivity. Moreover, a positive CBE is an abnormal clinical finding rather than a cancer diagnosis, so the available data cannot estimate positive predictive value, false-positive rates, cancer detection rates, or diagnostic sensitivity. The comparatively high monthly positivity observed during some low-volume months, including May 2020, further illustrates why individual monthly estimates should not be overinterpreted. Future routine reporting should link abnormal CBE findings to diagnostic assessment and final diagnosis to permit meaningful evaluation of program performance.

The revised interrupted time-series analysis also changes the interpretation of the temporal findings. Although cancer screening and early-detection services were disrupted during the COVID-19 pandemic in several LMIC settings [35,36], our analysis did not demonstrate a statistically significant immediate level change or change in monthly slope following March 2020. The confidence intervals around both estimates were wide, and sensitivity analyses using events per reporting facility, consistently reporting facilities, an alternative April 2020 interruption point, and an autoregressive specification yielded the same overall conclusion. We therefore cannot attribute the observed long-term pattern in Bangladesh to the pandemic on the basis of these data. Interpretation is additionally limited by the relatively short pre-intervention period, changing reporting completeness, and the observational nature of interrupted time-series analysis.

The NICRH registry data provide health-system context rather than outcomes of the CBE program. The finding that more than three-quarters of patients recorded in the 2018–2020 registry had received no prior treatment before presentation to NICRH is important in understanding cancer-care pathways in Bangladesh, but the registry population cannot be assumed to represent individuals included in the CBE dataset. The two datasets were not linked at the individual level, and the registry represents patients attending a tertiary cancer center. We therefore cannot determine whether individuals with positive CBE results completed referral, received diagnostic confirmation, initiated treatment, experienced stage shift, or had improved survival. These are critical outcomes for future evaluation of breast cancer early-detection services.

The findings have several practical implications. First, improving reporting completeness should be a prerequisite for using routine data to monitor population-level program performance. A stable facility reporting roster, routine completeness dashboards, and follow-up of missing facility-month reports could improve interpretability. Second, individual-level or privacy-preserving unique identifiers would allow repeat examinations to be distinguished from unique participants. Third, routine datasets should capture age, place of residence, reason for examination, referral destination, diagnostic completion, final diagnosis, and treatment initiation. Fourth, standardized training and quality-assurance indicators are needed to interpret differences in CBE positivity across facilities and regions. Integration with digital reporting platforms such as DHIS2 and stronger coordination with cancer registries could facilitate longitudinal monitoring [37–39].

These improvements are relevant to the WHO Global Breast Cancer Initiative, but its targets should not be interpreted as CBE screening-coverage benchmarks. The initiative aims, among other objectives, for at least 60% of invasive breast cancers to be diagnosed at stage I or II [17]. The present dataset contains neither stage at diagnosis nor person-level diagnostic outcomes and therefore cannot assess progress toward that target. Strengthening early detection must also occur alongside adequate diagnostic, referral, and treatment capacity; expanding examination activity without reliable downstream evaluation would not by itself demonstrate improved cancer outcomes [40–42].

A major strength of this study is the use of seven complete calendar years of facility-month program data covering a large national reporting network. The analysis includes the complete 2025 calendar year, explicit measurement of reporting completeness, facility-type analyses, denominator and high-volume-facility sensitivity analyses, fixed cohorts of consistently reporting facilities, and a seasonally adjusted interrupted time-series model with autocorrelation-robust inference. These additions allow the routine data to be characterized more transparently than in analyses based solely on aggregate examination totals.

Several limitations remain. Most importantly, reporting was incomplete throughout the study period and may have been differentially incomplete across facilities and geographic areas. The data represent examination events rather than confirmed unique individuals, so repeat examinations cannot be identified. District-level eligible populations were estimated using proportional allocation because age-specific district distributions were unavailable. The dataset did not include participant demographics, clinical indications, residence, examiner characteristics, quality-assurance measures, private-sector utilization, referral completion, diagnostic outcomes, cancer stage, treatment, or survival. Facility location therefore cannot be used to infer patient residence or population equity. The relatively short pre-March 2020 period and temporal changes in reporting also limit causal interpretation of the interrupted time-series analysis. Finally, NICRH registry indicators were contextual and not linked to CBE records at the individual level. Together, these limitations mean that the present study is best interpreted as an evaluation of reported national program activity and reporting performance, rather than an estimate of individual-level screening uptake or effectiveness.

## Conclusion

Routine CBE reporting in Bangladesh documented more than 3.19 million examination events from 2019 through 2025, with reported activity increasing through 2023 and declining thereafter. Geographic variation, concentration of activity among high-volume facilities, and persistently incomplete reporting were prominent features of the program. Because the data represent examination events from incompletely reporting facilities rather than identifiable individuals, the findings should not be interpreted as true population screening coverage or as evidence of screening effectiveness.

Improving reporting completeness, individual-level tracking, quality assurance, and linkage between abnormal CBE findings, diagnostic evaluation, and treatment is essential for reliable monitoring of breast cancer early-detection services in Bangladesh.

### Patient and public involvement

Patients and the public were not involved in the design, conduct, reporting, or dissemination of this research.

## Supporting information

S1 File Supplementary Appendix. Supplementary Tables A1–A6, including sensitivity analyses, coded geographic summaries, reporting completeness, consistently reporting facility analyses, and interrupted time-series results.
(DOCX)

S2 Data Coded district-level clinical breast examination activity, Bangladesh, 2019–2025. Dataset containing 448 district-year observations with coded district identifiers, reported CBE examination events, estimated eligible populations, and reported CBE examination rates. District names and the coding key are not included. Because some district codes may be inferable from exact aggregate values reported in the article, these data are described as coded rather than anonymous.
(XLSX)

S3 Data Monthly national clinical breast examination series used for interrupted time-series analysis, Bangladesh, 2019–2025. Monthly observed CBE examination events, reporting-facility counts, reporting completeness, events per reporting facility, and model-design variables underlying the interrupted time-series analyses and Fig 6.
(CSV)

S4 File STROBE checklist. Completed STROBE reporting checklist for this observational study.
(DOCX)

## References

[1] International Agency for Research on Cancer. Global Cancer Observatory. https://gco.iarc.fr/ Accessed 2025 November 24.

[2] Filho AM, Laversanne M, Ferlay J, Colombet M, Piñeros M, Znaor A, et al. The GLOBOCAN 2022 cancer estimates: Data sources, methods, and a snapshot of the cancer burden worldwide. Int J Cancer. 2025;156(7):1336–46. doi: 10.1002/ijc.35278 39688499

[3] Zhang Y, Ji Y, Liu S. Global burden of female breast cancer: new estimates in 2022, temporal trend, and projections to 2050. J Natl Cancer Cent. 2025;5(3):287–96. doi: 10.1016/j.jncc.2025.02.002 PMC12276554 40693239

[4] Lim YX, Lim ZL, Ho PJ, Li J. Breast cancer in Asia: incidence, mortality, early detection, mammography programs, and risk-based screening initiatives. Cancers (Basel). 2022;14(17):4218. doi: 10.3390/cancers14174218 36077752 PMC9454998

[5] Wang F, Liu S, Li J, Shi Y, Geng Z, Ji Y, et al. Burdens of breast cancer and projections for 2030 among women in Asia: findings from the 2021 global burden of disease study. Curr Oncol. 2025;32(5):267. doi: 10.3390/curroncol32050267 40422526 PMC12109881

[6] Mathur P, Sathishkumar K, Chaturvedi M. Cancer statistics, 2020: report from the National Cancer Registry Programme, India. JCO Glob Oncol. 2020;6:1063–75. doi: 10.1200/GO.20.00122 PMC7392737 32673076

[7] Afaya A, Ramazanu S, Bolarinwa OA. Health system barriers influencing timely breast cancer diagnosis and treatment among women in LMICs in Asia. BMC Health Services Research. 2022;22:1601. doi: 10.1186/s12913-022-08927-x PMC9805268 36587198

[8] Mubarik S, Sharma R, Hussain SR. Breast cancer mortality trends and predictions to 2030 in East and South Asia. Front Nutr. 2022;9:847920. doi: 10.3389/fnut.2022.847920 PMC8964109 35360680

[9] DeSantis CE, Bray F, Ferlay J, Lortet-Tieulent J, Anderson BO, Jemal A. International variation in female breast cancer incidence and mortality rates. Cancer Epidemiol Biomarkers Prev. 2015;24(10):1495–506. doi: 10.1158/1055-9965.EPI-15-0535 26359465

[10] National Institute of Cancer Research and Hospital. Hospital cancer registry 2018–2020 report. Dhaka: NICRH. 2022. https://nicrh.gov.bd/images/reports/41272-hbcr-2018-2020-report_compressed.pdf

[11] Huq MS, Acharya SC, Poudyal S. Cancer care and outreach in the SAARC region. Lancet Oncol. 2024;25(12):e650-62. doi: 10.1016/s1470-2045(24)00514-x 39637902

[12] Rahman MM, Islam MR, Rahman MS. Forgone healthcare and financial burden in Bangladesh. Health Econ Rev. 2022;12:5. doi: 10.1186/s13561-021-00348-6 PMC8751265 35006416

[13] Srinath A, van Merode F, Rao SV, Pavlova M. Barriers to cervical and breast cancer screening in LMICs: a systematic review. Health Policy Plan. 2023;38(4):509–27. doi: 10.1093/heapol/czac104 PMC10089064 36525529

[14] Ren W, Chen M, Qiao Y, Zhao F. Global guidelines for breast cancer screening: A systematic review. Breast. 2022;64:85–99. doi: 10.1016/j.breast.2022.04.003 35636342 PMC9142711

[15] Directorate General of Health Services. National cancer control strategy and plan of action 2009–2015. Dhaka: Directorate General of Health Services. 2008. https://www.iccp-portal.org/sites/default/files/plans/Publication_Cancer_Strategy.pdf

[16] World Health Organization. WHO supports early detection and control of cervical and breast cancer in Bangladesh. 2020. https://www.who.int/bangladesh/news/detail/10-11-2020-who-supports-early-detection-and-control-of-cervical-and-breast-cancer-in-bangladesh Accessed 2026 September 8.

[17] World Health Organization. WHO launches new roadmap on breast cancer. 2023. https://www.who.int/news/item/03-02-2023-who-launches-new-roadmap-on-breast-cancer Accessed 2025 December 4.

[18] National Institute of Cancer Research and Hospital. Surgical Oncology. https://nicrh.gov.bd/departments/surgical-oncology Accessed 2026 September 8.

[19] Khanna D, Sharma P, Budukh A, Khanna AK. Clinical breast examination as a screening tool in LMICs. Asia Pac J Clin Oncol. 2024;20(6):690–9. doi: 10.1111/ajco.14126 39342614

[20] International Agency for Research on Cancer. CanScreen5. https://canscreen5.iarc.fr/ Accessed 2025 June 12.

[21] Black E, Richmond R. Improving early detection of breast cancer in sub-saharan africa. Glob Health. 2019;15:3. doi: 10.1186/s12992-018-0446-6 PMC6325810 30621753

[22] Mittra I, Mishra GA, Dikshit RP. Two-decade follow-up of clinical breast examination screening in Mumbai. BMJ. 2021;372:n256. doi: 10.1136/bmj.n256 PMC7903383 33627312

[23] Ngan TT, Browne S, Goodwin M, Van Minh H, Donnelly M, O’Neill C. Cost-effectiveness of clinical breast examination screening programme among HER2-positive breast cancer patients: a modelling study. Breast Cancer. 2023;30(1):68–76. doi: 10.1007/s12282-022-01398-2 36028594 PMC9813013

[24] Mishra GA, Pimple SA, Mittra I, Badwe RA. Screening for breast cancer: cost-effective solutions for LMICs. Indian J Med Res. 2021;154(2):229–47. doi: 10.4103/ijmr.IJMR_2635_20 PMC9131750 34854430

[25] Pace LE, Uwihaye J de D, Mukandayisenga V, Fata AM, Rositch AF, Byiringiro J, et al. Early experience scaling up a breast cancer early detection initiative integrated with cervical cancer screening in Rwanda. BMJ Glob Health. 2025;10(10):e021235. doi: 10.1136/bmjgh-2025-021235 41120198 PMC12542583

[26] Dykstra M, Malone B, Lekuntwane O, Efstathiou J, Letsatsi V, Elmore S, et al. Impact of community-based clinical breast examinations in Botswana. JCO Glob Oncol. 2021;7:17–26. doi: 10.1200/GO.20.00231 33405960 PMC8081526

[27] Afaya A, Laari TT, Seidu AA, et al. Uptake of clinical breast examination in Lesotho. BMC Cancer. 2023;23:114. doi: 10.1186/s12885-023-10566-2 PMC9890772 36726101

[28] Ngan TT, Nguyen NTQ, Van Minh H, Donnelly M, O’Neill C. Effectiveness of clinical breast examination as a stand-alone screening modality. BMC Cancer. 2020;20:1070. doi: 10.1186/s12885-020-07521-w PMC7653771 33167942

[29] Government of Bangladesh. National cervical and breast cancer statistics dashboard. https://cancer-dashboard.mohfw.gov.bd/cervical-breast/ Accessed 2026 September 8.

[30] United Nations Department of Economic and Social Affairs. World population prospects 2022: methodology. New York: United Nations. 2022. https://www.un.org/development/desa/pd/content/World-Population-Prospects-2022

[31] Statistics and Informatics Division. Population and housing census 2022: preliminary report. Dhaka: Statistics and Informatics Division. 2022. https://sid.portal.gov.bd/

[32] Boulos S, Gadallah M, Neguib S, Essam E, Youssef A, Costa A, et al. Breast screening in the emerging world: high prevalence of breast cancer in Cairo. Breast. 2005;14(5):340–6. doi: 10.1016/j.breast.2005.07.002 16131468

[33] Rahimi F, Tolouei S, Ravankhah Z, Mohammadi-Sefiddashti F. Impact of clinical breast examination screening in the IraPEN project. Asian Pac J Cancer Prev. 2024;25(9):3151–7. doi: 10.31557/APJCP.2024.25.9.3151 PMC11700326 39342594

[34] Abuidris DO, Elsheikh A, Ali M, Musa H, Elgaili E, Ahmed AO, et al. Breast-cancer screening with trained volunteers in a rural area of Sudan: a pilot study. Lancet Oncol. 2013;14(4):363–70. doi: 10.1016/S1470-2045(12)70583-1 23375833

[35] Lucas E, Murillo R, Arrossi S. Quantifying the impact of the COVID-19 pandemic on cancer screening programmes in LMICs: case studies from six countries. eLife. 2023;12:e86527. doi: 10.7554/eLife.86527 PMC10188105 37191660

[36] Villain P, Carvalho AL, Lucas E, Mosquera I, Zhang L, Muwonge R, et al. Cross-sectional survey of the impact of the COVID-19 pandemic on cancer screening programs in selected low- and middle-income countries: Study from the IARC COVID-19 impact study group. Int J Cancer. 2021;149(1):97–107. doi: 10.1002/ijc.33500 33533501 PMC8014228

[37] Rahman MdM, Siddique MdKB, Rahim MMU. Cancer registry in Bangladesh: Challenges and Opportunities. IJTMRPH. 2024;8:e001. doi: 10.25259/ijtmrph_522

[38] International Agency for Research on Cancer. Global Initiative for Cancer Registry Development (GICR). Lyon: IARC; https://gicr.iarc.fr/ Accessed 2026 September 8.

[39] DHIS2. Expanding routine data use to improve health outcomes in Bangladesh. https://dhis2.org/expanding-routine-data-use-bangladesh/ Accessed 2026 September 8.

[40] Islam MR, Siddiqua SM, Mina S, et al. Cancer care in Bangladesh: urgent need for strategic reform. Discov Health Syst. 2025;4:96. doi: 10.1007/s44250-025-00283-x

[41] Ferdause J, Ahmed N, Rahman L. Understanding diagnostic delays among newly diagnosed breast cancer patients at a tertiary cancer centre in Bangladesh. Medicine (Baltimore). 2025;104(10):e41775. doi: 10.1097/MD.0000000000041775 PMC11902934 40068052

[42] Raghavan N, Jatoi I. Prioritizing mammography screening in developing countries: are we putting the cart before the horse? Ann Surg Oncol. 2024;31(3):1430–2. doi: 10.1245/s10434-023-14785-6 38112888

