# Supplementary material for: Reported clinical breast examination activity in Bangladesh, 2019–2025: Temporal trends, geographic variation, positivity, and reporting completeness
Source: PLoS One. 2026 Oct 29;21(10):e0361910. doi: 10.1371/journal.pone.0361910 (PMC13718965; doi:10.1371/journal.pone.0361910)
Supplement: Supplementary file 1 [file pone.0361910.s001.docx]

Appendix Table A1. Sensitivity analyses of reported CBE examination rates, Bangladesh, 2019–2025

| **Analysis** | **Mean rate (%)** | **Annual range (%)** | **Interpretation** |
| --- | --- | --- | --- |
| Main analysis, women aged 30–69 years | **1.3** | **0.3–2.4** | Primary analysis |
| Alternative denominator, women aged 30–64 years | **1.4** | **0.4–2.6** | Magnitude changed modestly; overall interpretation unchanged |
| Excluding highest-volume 5% of facilities | **0.9** | **0.2–1.8** | Demonstrated meaningful concentration of activity among high-volume facilities |
| Months with ≥60% national reporting completeness | Not estimable | Not estimable | No month met the prespecified 60% reporting threshold |

**Footnote:** CBE, clinical breast examination. Rates represent reported CBE examination events divided by the corresponding estimated eligible female population. The alternative denominator excluded women aged 65–69 years. The high-volume facility sensitivity excluded the 60 highest-volume facilities, representing approximately 5% of the 1,209-facility network. These facilities accounted for approximately 30.3% of all reported CBE events during the study period.

**Appendix Table A2.** Coded district-level reported CBE activity, Bangladesh, 2019–2025

The complete district-level dataset includes **64 districts across seven calendar years (448 district-year observations)**. To address data-sharing and privacy concerns, district names were removed from the supporting dataset and replaced with coded identifiers (D01–D64). The coding key is not included in the Supporting Information. Because exact aggregate counts are reported in the manuscript, some district codes may be inferable.

Each district-year record contains:

- Year
- Coded district identifier
- Reported CBE examination events
- Estimated eligible population of women aged 30–69 years
- Reported CBE examination rate

All 2025 values represent the complete **January–December 2025** calendar year.

**Footnote:** CBE, clinical breast examination. Reported CBE examination rates were calculated as reported CBE events divided by the estimated district-level population of women aged 30–69 years. District-level denominators were derived using proportional allocation from census-based female population distributions because district-specific age distributions were unavailable. Because facility reporting was incomplete and individual-level identifiers were unavailable, these rates should not be interpreted as true population screening coverage. The complete coded district-level dataset is provided separately as S2 Data.

Appendix Table A3. Division-level reported clinical breast examination activity, Bangladesh, 2019, 2023, and 2025

| **Year** | **Division** | **Reported CBE**  **events (n)** | **Eligible women aged**  **30–69 years (N)** | **Reported CBE**  **examination**  **rate (%)** |
| --- | --- | --- | --- | --- |
| 2019 | Barishal | 3,747 | 1,839,189 | 0.2 |
| 2019 | Chattogram | 16,579 | 6,774,721 | 0.2 |
| 2019 | Dhaka | 45,340 | 8,576,309 | 0.5 |
| 2019 | Khulna | 15,740 | 3,469,346 | 0.5 |
| 2019 | Mymensingh | 1,949 | 2,461,332 | <0.1 |
| 2019 | Rajshahi | 19,328 | 4,052,462 | 0.5 |
| 2019 | Rangpur | 10,171 | 3,504,162 | 0.3 |
| 2019 | Sylhet | 1,206 | 2,229,874 | <0.1 |
| 2023 | Barishal | 49,452 | 1,932,970 | 2.6 |
| 2023 | Chattogram | 166,912 | 7,120,168 | 2.3 |
| 2023 | Dhaka | 225,852 | 9,013,621 | 2.5 |
| 2023 | Khulna | 114,079 | 3,646,250 | 3.1 |
| 2023 | Mymensingh | 40,118 | 2,586,836 | 1.6 |
| 2023 | Rajshahi | 121,948 | 4,259,100 | 2.9 |
| 2023 | Rangpur | 81,592 | 3,682,841 | 2.2 |
| 2023 | Sylhet | 44,639 | 2,343,576 | 1.9 |
| 2025 | **Barishal** | **24,856** | **2,046,277** | **1.2** |
| 2025 | **Chattogram** | **65,180** | **7,537,537** | **0.9** |
| 2025 | **Dhaka** | **114,483** | **9,541,980** | **1.2** |
| 2025 | **Khulna** | **65,684** | **3,859,986** | **1.7** |
| 2025 | **Mymensingh** | **18,183** | **2,738,471** | **0.7** |
| 2025 | **Rajshahi** | **68,189** | **4,508,759** | **1.5** |
| 2025 | **Rangpur** | **36,151** | **3,898,721** | **0.9** |
| 2025 | **Sylhet** | **24,562** | **2,480,952** | **1.0** |

**Footnote:** CBE, clinical breast examination. Rates represent reported CBE examination events divided by the estimated population of women aged 30–69 years within each division. All 2025 values represent January–December 2025. Because facility reporting was incomplete and individual-level identifiers were unavailable, these values should not be interpreted as true population screening coverage.

Appendix Table A4. Annual reporting completeness of the national CBE program, Bangladesh, 2019–2025

| **Year** | **Submitted facility-month reports** | **Expected facility-month reports** | **Mean reporting completeness (%)** | **Monthly range (%)** |
| --- | --- | --- | --- | --- |
| 2019 | 1,759 | 14,508 | 12.1 | 8.2–16.6 |
| 2020 | 2,557 | 14,508 | 17.6 | 4.5–28.2 |
| 2021 | 4,510 | 14,508 | 31.1 | 25.8–39.3 |
| 2022 | 5,370 | 14,508 | 37.0 | 34.6–40.8 |
| 2023 | 6,088 | 14,508 | 42.0 | 40.7–43.4 |
| 2024 | 6,129 | 14,508 | 42.2 | 40.3–43.4 |
| 2025 | 5,765 | 14,508 | 39.7 | 36.9–41.4 |

**Footnote:** Reporting completeness was defined as the number of submitted facility-month reports divided by the expected number of reports from 1,209 location-specific eligible facilities. No month during the study period reached 60% national reporting completeness. A sensitivity analysis allowing 1,216 eligible facilities for October–December 2025 produced a negligible change in the 2025 estimate. Reporting completeness improved substantially after 2020 but remained incomplete throughout the study period.

Appendix Table A5. Sensitivity analysis restricted to consistently reporting facilities, Bangladesh, 2019–2025

| **Year** | **Strict cohort: 48 facilities, CBE events (n)** | **Moderate cohort: 118 facilities, CBE events (n)** |
| --- | --- | --- |
| 2019 | 37,334 | 71,466 |
| 2020 | 35,144 | 75,630 |
| 2021 | 75,362 | 165,022 |
| 2022 | 102,137 | 221,299 |
| 2023 | **121,515** | **260,265** |
| 2024 | 89,679 | 191,241 |
| 2025 | **72,392** | **157,461** |

| **Year** | **Strict submitted reports** | **Strict events/report** | **Moderate submitted reports** | **Moderate events/report** |
| --- | --- | --- | --- | --- |
| 2019 | 513 | 72.8 | 1,069 | 66.9 |
| 2020 | 501 | 70.1 | 1,093 | 69.2 |
| 2021 | 563 | 133.9 | 1,346 | 122.6 |
| 2022 | 574 | 177.9 | 1,405 | 157.5 |
| 2023 | 572 | 212.4 | 1,408 | 184.8 |
| 2024 | 574 | 156.2 | 1,402 | 136.4 |
| 2025 | 570 | 127.0 | 1,382 | 113.9 |

**Footnote:** Strict cohort: facilities reporting in at least 9 months of every year; moderate cohort: at least 6 months. The first panel shows CBE events; the second shows submitted facility-month reports and events per submitted report. From 2023 to 2025, reports were nearly unchanged (strict: 572 to 570; moderate: 1,408 to 1,382), while events per report declined (212.4 to 127.0; 184.8 to 113.9).

Appendix Table A6. Interrupted time-series analysis of monthly reported CBE activity, Bangladesh, 2019–2025

| **Model** | **Parameter** | **Estimate** | **95% CI** | **P value** |
| --- | --- | --- | --- | --- |
| **Primary model: all monthly CBE events, seasonality + HAC(12)** | Pre-intervention monthly trend | 762 events/month | −303 to 1,827 | 0.161 |
|  | Immediate level change at March 2020 | 14,416 events | −7,438 to 36,269 | 0.196 |
|  | Post-intervention slope change | −375 events/month | −1,673 to 923 | 0.571 |
| **Sensitivity: CBE events per reporting facility, corrected facility identity, seasonality + HAC(12)** | Pre-intervention monthly trend | 0.50 events/reporting facility/month | −1.48 to 2.48 | 0.620 |
|  | Immediate level change at March 2020 | 11.52 events/reporting facility | −36.30 to 59.34 | 0.637 |
|  | Post-intervention slope change | −0.12 events/reporting facility/month | −2.41 to 2.17 | 0.916 |
| **Sensitivity: fixed cohort of 48 consistently reporting facilities, seasonality + HAC(12)** | Pre-intervention monthly trend | 199 events/month | −6 to 403 | 0.057 |
|  | Immediate level change at March 2020 | 580 events | −2,703 to 3,864 | 0.729 |
|  | Post-intervention slope change | −151 events/month | −378 to 75 | 0.190 |
| **Sensitivity: April 2020 interruption, seasonality + HAC(12)** | Pre-intervention monthly trend | 593 events/month | −336 to 1,523 | 0.211 |
|  | Immediate level change at April 2020 | 17,120 events | −3,821 to 38,060 | 0.109 |
|  | Post-intervention slope change | −233 events/month | −1,468 to 1,002 | 0.712 |
| **Sensitivity: March 2020 with AR(1) errors** | Pre-intervention monthly trend | 762 events/month | −9,488 to 11,012 | 0.884 |
|  | Immediate level change at March 2020 | 14,416 events | −6,650 to 35,482 | 0.180 |
|  | Post-intervention slope change | −375 events/month | −10,964 to 10,214 | 0.945 |

**Footnote:** CBE, clinical breast examination; CI, confidence interval; HAC, heteroskedasticity- and autocorrelation-consistent standard errors; AR(1), first-order autoregressive errors. The primary segmented regression included calendar-month fixed effects to account for seasonality and Newey-West/HAC standard errors with 12 monthly lags. March 2020 was specified as the primary interruption point. Sensitivity analyses used corrected location-specific reporting-facility identity, an April 2020 interruption point, and an AR(1) error specification. Neither the immediate level change nor the post-intervention slope change was statistically significant in any model.

**Appendix Notes**

- Values shown as <0.1% indicate non-zero rates that round to 0.0% when expressed to one decimal place.
- All 2025 analyses represent the complete January–December 2025 calendar year.
- Reported CBE examination rates are based on recorded CBE events and should not be interpreted as true population screening coverage because facility reporting was incomplete and individual-level deduplication was unavailable.
