# Supplementary material for: Reported clinical breast examination activity in Bangladesh, 2019–2025: Temporal trends, geographic variation, positivity, and reporting completeness
Source: PLoS One. 2026 Oct 29;21(10):e0361910. doi: 10.1371/journal.pone.0361910 (PMC13718965; doi:10.1371/journal.pone.0361910)
Supplement: Supplementary file 2 [file pone.0361910.s004.docx]

**STROBE Checklist - Observational Study**

Manuscript title: Reported clinical breast examination activity in Bangladesh, 2019–2025: temporal trends, geographic variation, positivity, and reporting completeness

| **Section/Topic** | **Item No.** | **STROBE recommendation** | **Reported in Manuscript (Page/Section)** |
| --- | --- | --- | --- |
| Title and abstract | 1 | Indicate the study design with a commonly used term and provide an informative, balanced summary of what was done and found. | Title, p. 1; Abstract, pp. 2–3; Methods – Study design and setting, p. 5. |
| Background/rationale | 2 | Explain the scientific background and rationale for the investigation. | Introduction, pp. 3–4. |
| Objectives | 3 | State specific objectives, including any prespecified hypotheses where applicable. | Introduction – final paragraph, p. 4. |
| Study design | 4 | Present key elements of the study design early in the paper. | Methods – Study design and setting, p. 5. |
| Setting | 5 | Describe the setting, locations, and relevant dates, including periods of data collection and follow-up where applicable. | Methods – Study design and setting and Data sources and reporting structure, pp. 5–6. Study period: Jan 2019–Dec 2025; data-access dates: lines 131–133. |
| Participants | 6 | Describe eligibility criteria and the sources and methods of selection. For matched studies, give matching criteria and numbers. | Methods – Study design and setting, p. 5. The analysis used a prespecified network of 1,209 eligible reporting facilities. Individual participant recruitment and matching were not applicable. |
| Variables | 7 | Clearly define outcomes, exposures, predictors, potential confounders, and effect modifiers. Give diagnostic criteria if applicable. | Methods – Outcomes, pp. 6–7; Statistical analysis, pp. 7–8. |
| Data sources / measurement | 8 | For each variable of interest, give sources of data and details of assessment or measurement. | Methods – Data sources and reporting structure and Population denominators, pp. 5–6. |
| Bias | 9 | Describe efforts to address potential sources of bias. | Methods – Population denominators and Statistical analysis, pp. 6–8; Discussion, pp. 12–15. Reporting completeness, denominator assumptions, fixed reporting cohorts, and sensitivity analyses are addressed. |
| Study size | 10 | Explain how the study size was arrived at. | Methods – Study design and setting, p. 5. All available records from the prespecified 1,209-facility network during the study period were included; no sample-size calculation was performed. |
| Quantitative variables | 11 | Explain how quantitative variables were handled in the analyses, including any groupings and why they were chosen. | Methods – Population denominators, Outcomes, and Statistical analysis, pp. 6–8. Annual/monthly summaries, age-denominator definitions, facility categories, and prespecified sensitivity thresholds are described. |
| Statistical methods | 12 | Describe all statistical methods, including subgroup analyses, handling of missing data, sensitivity analyses, and methods used to address confounding or sampling where applicable. | Methods – Data sources and reporting structure, Outcomes, and Statistical analysis, pp. 5–8. Missing facility-month reports were not imputed; geographic/facility analyses, Spearman correlation, fixed cohorts, ITS specification, and sensitivity analyses are described. Participant-level confounder adjustment and loss-to-follow-up analysis were not applicable to the aggregate dataset. |
| Participants / observations | 13 | Report numbers at each stage of the study and reasons for non-participation where applicable; consider a flow diagram. | Results – National reported CBE activity, p. 8: 32,178 facility-month records and 3,193,080 reported CBE events. Individual participant flow was not available because the data were aggregate facility-month records. |
| Descriptive data | 14 | Give characteristics of study participants or observations, information on missing data, and follow-up time where applicable. | Methods, pp. 5–8; Results, pp. 8–10. Geographic distribution, facility type, positivity, and reporting completeness are reported. Participant-level demographics and follow-up were unavailable and are stated as limitations. |
| Outcome data | 15 | Report numbers of outcome events or summary measures over time. | Results – National activity, Geographic variation, CBE positivity, and Reporting completeness, pp. 8–10; Tables 1–5. |
| Main results | 16 | Give unadjusted and adjusted estimates as applicable, their precision, and relevant category boundaries; translate relative risk into absolute risk when useful. | Results, pp. 8–11. Annual and geographic rates and positivity are reported; ITS estimates include 95% CIs and P values. Adjusted participant-level effect estimates and risk translation were not applicable. |
| Other analyses | 17 | Report other analyses performed, such as subgroup, interaction, and sensitivity analyses. | Results – Sensitivity analyses and Interrupted time-series analysis, pp. 10–11; Appendix Tables A1, A5, and A6. |
| Key results | 18 | Summarize key results with reference to study objectives. | Discussion – opening paragraph, p. 12. |
| Limitations | 19 | Discuss limitations, including direction and magnitude of potential bias or imprecision. | Discussion, pp. 12–15, especially the limitations paragraph on p. 15. |
| Interpretation | 20 | Give a cautious overall interpretation considering objectives, limitations, multiplicity of analyses, results from similar studies, and other relevant evidence. | Discussion, pp. 12–15. |
| Generalisability | 21 | Discuss the generalisability (external validity) of the study results. | Discussion, pp. 13–15. The manuscript limits inference to reported program activity and explains why findings should not be interpreted as true population coverage, patient residence, or clinical effectiveness. |
| Funding | 22 | Give the source of funding and the role of funders. | Funding, p. 16: no specific funding was received for this study. |

*Note: This study used aggregate facility-month program data rather than individual participant records. Participant-level recruitment, demographics, matching, follow-up, and individual-level confounder adjustment were therefore not available or not applicable. Where a STROBE item is not applicable for this data structure, this is stated explicitly above.*
