# Supplementary material for: Reported clinical breast examination activity in Bangladesh, 2019–2025: Temporal trends, geographic variation, positivity, and reporting completeness
Source: PLoS One. 2026 Oct 29;21(10):e0361910. doi: 10.1371/journal.pone.0361910 (PMC13718965; doi:10.1371/journal.pone.0361910)
Supplement: Supplementary file 3 [file pone.0361910.s005.docx]

Response to the Academic Editor and Reviewers

**Manuscript ID: PONE-D-26-23766**

Revised title: Reported clinical breast examination activity in Bangladesh, 2019–2025: temporal trends, geographic variation, positivity, and reporting completeness

**Dear Academic Editor and Reviewers,**

Thank you for the careful and constructive evaluation of our manuscript. We have substantially revised the manuscript to address the concerns raised by the Academic Editor and all five reviewers. The revised analysis now uses complete January through December 2025 data and consistently treats the routine records as reported clinical breast examination events rather than unique women screened. We have also made reporting completeness central to the interpretation, replaced the rural versus urban analysis with a facility-type description, reanalyzed the interrupted time series with seasonality and autocorrelation addressed, expanded sensitivity analyses, strengthened the description of data structure and reporting, clarified the limited role of the NICRH registry data, and revised the terminology and conclusions throughout.

The principal revisions are summarized below:

- The title, abstract, results, discussion, tables, figures, and conclusion now use “reported CBE activity,” “reported CBE events,” and “reported CBE examination rate” rather than presenting the estimates as true national screening coverage.
- Complete October through December 2025 program data were incorporated, so all seven study years now represent complete calendar years.
- The Methods now state that the data are aggregate facility-month records, that individual identifiers were unavailable, and that repeat examinations could not be deduplicated.
- The reporting structure is described as electronic monthly reporting to the national program database, with the locally collected fields specified. The data-access dates for the original and updated exports are also stated.
- Reporting completeness is explicitly defined, reported annually and geographically, and incorporated into interpretation. Fixed cohorts of consistently reporting facilities were added to assess whether temporal patterns were driven by changes in the reporting set.
- The prior rural versus urban comparison was removed because facility location is not equivalent to participant residence. It was replaced by a descriptive analysis of reported activity by facility type.
- The interrupted time-series model was re-specified with calendar-month fixed effects and Newey-West heteroskedasticity- and autocorrelation-consistent standard errors with 12 monthly lags. Sensitivity analyses were added, and the revised model does not show a statistically significant immediate or sustained change associated with March 2020.
- CBE positivity is now described as the proportion of reported examinations with an abnormal CBE finding, not as cancer detection. The limitations created by missing age, symptoms, risk factors, examiner quality measures, referral completion, and diagnostic confirmation are stated explicitly.
- NICRH registry indicators are presented only as contextual tertiary-center data and are explicitly not linked to the CBE records or interpreted as national outcomes of the CBE program.
- Supporting Information was revised to include anonymized geographic data, the monthly national series underlying the interrupted time-series analysis, revised sensitivity analyses, and the STROBE checklist. Supporting Information captions are provided at the end of the manuscript.
- The reference list and in-text citations were reviewed for relevance, corrected where necessary, and renumbered in order of first appearance.

# Response to Journal Requirements

**Comment:** 1. Please ensure that the manuscript meets PLOS ONE style requirements, including file naming.

**Response:** We reviewed the revised manuscript for PLOS ONE presentation and citation style. The clean revised manuscript, Supporting Information files, figures, STROBE checklist, and this point-by-point response are being prepared as separate submission files with clear descriptive names.

**Location in revised manuscript:** Throughout the revised submission files.

**Comment:** 2. The Data Availability Statement should include a DOI/accession number or direct link to each database.

**Response:** The Data Availability Statement now provides the direct link to the Government of Bangladesh national cervical and breast cancer statistics dashboard. The processed data supporting the analyses are also provided as Supporting Information, including anonymized geographic summaries and the monthly national interrupted time-series dataset.

**Location in revised manuscript:** Data availability statement; Supporting Information captions.

**Comment:** 3. The ethics statement should appear only in the Methods section.

**Response:** The ethics statement now appears only in the Methods section. Duplicate ethics wording outside the Methods was removed.

**Location in revised manuscript:** Methods, Ethics statement.

**Comment:** 4. Supporting Information captions should be included at the end of the manuscript.

**Response:** Captions for S1 File, S2 Data, S3 Data, and S4 Checklist have been added at the end of the revised manuscript.

**Location in revised manuscript:** End of manuscript, Supporting Information.

**Comment:** 5. Potentially identifying location data in the Supporting Information should be removed or anonymized, and hidden columns should not be used.

**Response:** We removed direct geographic identifiers from the public Supporting Information. Districts are represented by stable anonymized codes, and the coding key is not provided. Division-level Supporting Information uses anonymized division codes. The identifying columns were removed rather than hidden.

**Location in revised manuscript:** Supporting Information, S1 File and S2 Data.

**Comment:** 6. Suggested citations should be evaluated for relevance rather than cited automatically.

**Response:** We reviewed the reference list against the revised text, removed references that no longer supported a retained claim, corrected citation details where necessary, and retained only references that are relevant to the final manuscript.

**Location in revised manuscript:** Reference list and in-text citations throughout.

# Response to the Academic Editor

**Comment:** The main issues concern low reporting completeness, uncertainty about whether the records represent unique women or examinations, partial-year 2025 data, the rural versus urban comparison, interrupted time-series analysis, terminology such as “screening” and “national coverage,” and numerical and methodological consistency.

**Response:** We addressed each of these issues directly. Reporting completeness is now a central result and limitation; the numerator is explicitly defined as reported examination events rather than unique women; complete October through December 2025 data were added; the rural versus urban analysis was removed and replaced by facility type; the interrupted time-series analysis was re-specified and interpreted cautiously; terminology was changed throughout to emphasize reported CBE activity rather than true national coverage; and all principal numbers were rechecked across the abstract, text, tables, figures, and Supporting Information. The revised title reflects this reframing.

**Location in revised manuscript:** Title; Abstract; Methods; Results; Discussion; Conclusion; Tables 1–6; Figs 1–6; Supporting Information.

# Reviewer #1

## Major concerns

**Comment:** 1. Low reporting completeness may make the original coverage estimates unreliable. The analysis should be reframed, imputed, or restricted to sufficiently complete periods.

**Response:** We agree that incomplete reporting is the key limitation. We chose the reviewer’s recommended reframing approach rather than imputation because the aggregate data do not provide a defensible basis for assumptions about the missing facility-month observations. The manuscript now consistently describes the numerator as reported CBE examination events and the population metric as a reported CBE examination rate rather than true screening coverage. Reporting completeness is explicitly defined and presented by year and geographically for 2025, and no month is presented as meeting a 60% national completeness threshold. We also added fixed-cohort analyses of 48 facilities reporting in at least 9 months of every year and 118 facilities reporting in at least 6 months of every year. These cohorts also show a decline after the 2023 peak, while the manuscript remains explicit that incomplete reporting prevents precise estimation of population-level change. The revised interrupted time-series analysis and its sensitivity analyses no longer support a definitive COVID-associated decline.

**Location in revised manuscript:** Title; Abstract; Methods, Outcomes and Statistical analysis; Results, Reporting completeness and Sensitivity analyses; Discussion; Conclusion; Appendix Tables A1, A4, A5, and A6.

**Comment:** 2. District denominators rely on proportional allocation, and the 30–69-year denominator may not correspond to a population-based screening cohort.

**Response:** We clarified the denominator construction and its assumptions. National denominators are based on age- and sex-specific United Nations estimates for women aged 30–69 years. District denominators are obtained by proportionally allocating the national eligible population using census-based female population distributions because district-specific age distributions were unavailable. This assumption is now stated explicitly and district rates are described as approximate. We also retained a 30–64-year denominator sensitivity analysis. Because the numerator represents reported examination events rather than a deduplicated population cohort, the metric is no longer interpreted as true population screening coverage.

**Location in revised manuscript:** Methods, Population denominators and Outcomes; Results, Sensitivity analyses; Discussion, limitations; Appendix Table A1.

**Comment:** 3. The rural versus urban findings are difficult to interpret and may reflect facility distribution, denominator artifacts, or private-sector use.

**Response:** We agree. The prior rural versus urban analysis has been removed entirely because the available data identify the reporting facility, not the participant’s residence, and valid rural- and urban-specific population denominators were not available. It has been replaced by a descriptive analysis of reported CBE activity by facility type. The revised Discussion explicitly states that facility type cannot be interpreted as patient residence and notes that care received through private or other facilities outside the program dataset is not captured.

**Location in revised manuscript:** Methods, Outcomes; Results, Distribution of activity by facility type; Discussion; Table 4 and Fig 4.

**Comment:** 4. The approximately 1% positivity rate should be interpreted cautiously because quality assurance, case mix, demographics, diagnostic follow-up, and false-positive information are unavailable.

**Response:** We agree and substantially revised this interpretation. A positive CBE is now defined as a reported abnormal examination finding, not a confirmed cancer diagnosis. The Discussion states that the dataset lacks participant age, breast cancer risk factors, symptoms, examiner training, quality-assurance measures, referral completion, and diagnostic confirmation. We therefore do not estimate positive predictive value, false-positive rates, cancer detection rates, or diagnostic sensitivity. Comparisons with other settings are explicitly described as cautious.

**Location in revised manuscript:** Methods, Outcomes; Results, CBE positivity; Discussion; Table 5 and Fig 5.

**Comment:** 5. NICRH registry data are not linked to the CBE cohort and cannot establish treatment, stage, or survival outcomes.

**Response:** We agree. The revised manuscript now describes the NICRH registry data only as contextual tertiary-center indicators. It explicitly states that the two datasets were not linked at the individual level and that the registry cannot be used to estimate diagnostic confirmation, referral completion, treatment, stage, or survival among people represented in the CBE program.

**Location in revised manuscript:** Methods, Data sources and reporting structure; Results, Contextual NICRH registry indicators; Discussion; Table 6.

## Minor concerns

**Comment:** 1. The Methods should define reporting completeness, describe cleaning procedures, and fully specify the interrupted time-series analysis.

**Response:** Reporting completeness is now defined as submitted facility-month reports divided by expected facility-month reports from the prespecified 1,206-facility network. The Methods describe harmonization of administrative and facility-name variants, standardization of facility categories, duplicate checks, required-field checks, and arithmetic checks of positive, negative, and total counts. Missing facility-month reports were not imputed. The interrupted time-series specification now includes continuous time, a post-March 2020 indicator, time after the interruption, calendar-month fixed effects, and Newey-West heteroskedasticity- and autocorrelation-consistent standard errors with 12 monthly lags. Additional sensitivity models are also described.

**Location in revised manuscript:** Methods, Data sources and reporting structure; Outcomes; Statistical analysis.

**Comment:** 2. Tables and figures duplicate information, Figure 1 does not show monthly ITS data, and the rural versus urban table uses unsuitable denominators.

**Response:** The rural versus urban table and analysis were removed. Figure 6 was revised specifically for the interrupted time series and now displays observed monthly values, fitted values, the pre-intervention counterfactual trajectory, and the March 2020 intervention point. We considered consolidation of the remaining tables and figures, but retained them because they address distinct reviewer-requested domains: annual national activity, geographic extremes, facility type, positivity, registry context, and interrupted time-series results. Detailed geographic and sensitivity material is placed in Supporting Information rather than expanded further in the main text.

**Location in revised manuscript:** Table 4 and Fig 4; Fig 6; Supporting Information.

**Comment:** 3. The WHO Global Breast Cancer Initiative 60% target was compared incorrectly with CBE coverage.

**Response:** We corrected this conceptual issue. The revised Discussion states that the WHO target refers to the proportion of invasive breast cancers diagnosed at stage I or II and should not be interpreted as a CBE coverage benchmark. The manuscript also states that the present data do not contain stage at diagnosis and therefore cannot assess progress toward that target.

**Location in revised manuscript:** Introduction; Discussion, WHO Global Breast Cancer Initiative paragraph.

**Comment:** 4. The reference list contains questionable access dates and a potentially misused Raghavan and Jatoi citation.

**Response:** We reviewed and cleaned the reference list, removed unused references, corrected citation details, and standardized web-reference access information. The Raghavan and Jatoi article is no longer used as evidence for a specific claim about current treatment infrastructure. It is retained only in the broader statement that expansion of early-detection activity should occur alongside adequate diagnostic, referral, and treatment capacity, which is consistent with the article’s central argument about sequencing screening investment in resource-constrained settings.

**Location in revised manuscript:** Reference list; Discussion, paragraph on diagnostic, referral, and treatment capacity.

**Comment:** 5. Policy recommendations are generic and not clearly derived from the findings.

**Response:** We revised the recommendations to follow directly from the observed limitations. The Discussion now specifies a stable reporting roster, routine completeness dashboards, follow-up of missing facility-month reports, privacy-preserving unique identifiers, collection of age and residence, reason for examination, referral destination, diagnostic completion, final diagnosis and treatment initiation, standardized training and quality-assurance indicators, and integration with digital reporting and cancer registries. The previous catalogue of international registry examples was removed.

**Location in revised manuscript:** Discussion, practical implications paragraph.

# Reviewer #2

**Comment:** Title: remove “COVID-19” for clarity.

**Response:** The title has been changed and no longer includes “COVID-19.” The revised title emphasizes reported CBE activity, geographic variation, positivity, and reporting completeness.

**Location in revised manuscript:** Title page.

**Comment:** Short title: replace the acronym CBE with clearer wording.

**Response:** The running head is now “Reported breast examination activity in Bangladesh,” without the acronym CBE.

**Location in revised manuscript:** Title page.

**Comment:** Correspondence: use an institutional email address whenever possible.

**Response:** We appreciate this suggestion. The correspondence address currently used for the submission has been retained in the manuscript. We will update it to an institutional address if one is available and required by the journal.

**Location in revised manuscript:** Title page, correspondence details.

**Comment:** Introduction: provide more context on mammographic screening, patient management, referral centers, and breast units in Bangladesh.

**Response:** The Introduction now states that the Bangladesh National Cancer Control Strategy and Plan of Action considered population mammographic screening impractical in the national context at that time and proposed CBE as a feasible early-detection approach. We also describe expansion of public-sector CBE services through district hospitals and upazila health complexes, explain that abnormal examinations require diagnostic evaluation and referral, and note that NICRH includes a dedicated breast surgical unit and diagnostic and oncologic services. We avoided adding adherence estimates or national mammography coverage figures that are not supported by the routine dataset used in this study.

**Location in revised manuscript:** Introduction, Bangladesh program context paragraph.

**Comment:** Language and terminology, including plural wording and standardization of “programme” to “program.”

**Response:** The manuscript was reviewed for these language issues. “Program” is used consistently for the Bangladesh service and the wording around high-income settings and early diagnosis was revised for clarity. Publication titles in the reference list retain their original wording where applicable.

**Location in revised manuscript:** Throughout the manuscript.

**Comment:** Methods: explain how centers report data, how often reporting occurs, and what information is collected locally.

**Response:** The Methods now state that participating facilities reported routine program data electronically on a monthly basis to the national program database. The available fields are listed, including reporting period, division, district, upazila, facility, facility category, examination type, positive examinations, negative examinations, and total examinations. The dates on which the original and updated data exports were accessed for research are also reported.

**Location in revised manuscript:** Methods, Data sources and reporting structure.

**Comment:** Explain the term “upazila.”

**Response:** An upazila is now defined as a subdistrict administrative unit, and an upazila health complex is described as a public-sector facility serving that level of the health system.

**Location in revised manuscript:** Methods, Study design and setting.

**Comment:** Discuss the marked decrease in assessments observed in 2023 and possible causes.

**Response:** The revised complete-year analysis shows that 2023 was the peak year, followed by decreases in 2024 and 2025. We therefore corrected the temporal description rather than attributing a decrease to 2023. Fixed-cohort analyses show that the post-2023 decline also occurred among consistently reporting facilities, but the aggregate routine data do not identify the causal reason for the decline. We therefore avoid unsupported causal explanations and emphasize the limits imposed by incomplete reporting.

**Location in revised manuscript:** Results, National reported CBE activity and Sensitivity analyses; Discussion.

**Comment:** Discuss whether the rural versus urban difference could reflect mammography use in urban areas.

**Response:** The rural versus urban comparison was removed because the source data do not contain patient residence and did not support valid rural- versus urban-population coverage estimates. The revised manuscript instead describes activity by facility type and notes that use of private or other services not captured by the program could affect observed patterns.

**Location in revised manuscript:** Methods; Results, Distribution of activity by facility type; Discussion; Table 4 and Fig 4.

**Comment:** Define NICRH at first mention.

**Response:** NICRH is now written in full as the National Institute of Cancer Research and Hospital at first mention.

**Location in revised manuscript:** Introduction.

**Comment:** Use thousands separators and improve table alignment.

**Response:** Thousands separators are now used consistently in the principal tables, and the revised tables were reformatted for clearer alignment and readability.

**Location in revised manuscript:** Tables 1–6.

**Comment:** Strengthen the statistical analysis beyond descriptive summaries.

**Response:** The revised analysis includes a Spearman correlation between district reporting completeness and reported CBE examination rate in 2025, fixed cohorts of consistently reporting facilities, the high-volume-facility sensitivity analysis, denominator sensitivity analysis, and a re-specified interrupted time-series model with seasonality and autocorrelation addressed. These analyses are reported alongside the descriptive results.

**Location in revised manuscript:** Methods, Statistical analysis; Results, Reporting completeness, Sensitivity analyses, and Interrupted time-series analysis; Appendix Tables A1, A5, and A6.

# Reviewer #3

**Comment:** Low reporting completeness means the findings should be framed as reported program activity rather than complete national coverage, and consistent reporters should be analyzed where possible.

**Response:** We agree. This reframing is now applied throughout the manuscript. Reporting completeness is presented by year and geographically, and fixed cohorts of consistently reporting facilities were added. The abstract and conclusion explicitly state that the findings describe reported activity and should not be interpreted as true national screening coverage.

**Location in revised manuscript:** Title; Abstract; Methods; Results; Discussion; Conclusion; Appendix Tables A4 and A5.

**Comment:** Clarify whether the numerator represents unique women or examinations.

**Response:** The Methods now state explicitly that the data are aggregate facility-month records without individual identifiers, so repeat examinations cannot be identified. The analytic numerator is therefore reported CBE examination events, not unique women screened. This terminology is used throughout the revised manuscript.

**Location in revised manuscript:** Abstract; Methods, Study design and setting; Tables and figure captions; Discussion; Conclusion.

**Comment:** The rural versus urban analysis is not valid as a population equity comparison.

**Response:** We removed the rural versus urban analysis and replaced it with a descriptive facility-type analysis. The revised text explicitly states that facility location is not patient residence and cannot be used to infer population equity.

**Location in revised manuscript:** Methods; Results, Distribution of activity by facility type; Discussion; Table 4 and Fig 4.

**Comment:** The original 2025 data were partial year and should not be compared directly with full years.

**Response:** We obtained and incorporated October through December 2025 data. The revised study therefore covers seven complete calendar years from January 2019 through December 2025. All 2025 national, geographic, positivity, completeness, and interrupted time-series results were recalculated using the full year.

**Location in revised manuscript:** Abstract; Methods, Data sources and reporting structure; Results; Tables 1–5; Figs 1–6; Supporting Information.

**Comment:** The interrupted time-series model should be fully described and should not be presented as definitive evidence of a sustained or causal COVID-19 effect.

**Response:** We reanalyzed the monthly series using a segmented regression with continuous time, a post-March 2020 indicator, time after the interruption, calendar-month fixed effects, and Newey-West heteroskedasticity- and autocorrelation-consistent standard errors with 12 monthly lags. The revised immediate level change and slope change are both statistically non-significant. Sensitivity analyses using events per reporting facility, a fixed cohort, an April 2020 interruption point, and an autoregressive specification lead to the same overall conclusion. The manuscript now explicitly states that the analysis does not provide statistically conclusive evidence of an immediate or sustained COVID-associated change and does not support causal attribution.

**Location in revised manuscript:** Abstract; Methods, Statistical analysis; Results, Interrupted time-series analysis; Discussion; Fig 6; Appendix Table A6.

**Comment:** Correct numerical inconsistencies across the manuscript and Supporting Information.

**Response:** We performed a full numerical consistency review after incorporating the complete 2025 data. The previous rural versus urban difference and related sign issue were removed with that analysis. The division and district extremes were corrected, including the 2023 Sunamganj value, and the high-volume-facility exclusion is now described as a meaningful reduction from approximately 1.3% to 0.9%. The abstract, text, tables, figures, and Supporting Information were updated to the same final analysis.

**Location in revised manuscript:** Results; Tables 1–6; Figs 1–6; Supporting Information.

**Comment:** Revise clarity, grammar, terminology, and numerical consistency throughout.

**Response:** The manuscript was revised throughout for standard English, consistent use of “program,” cautious use of “screening,” consistent event-based terminology, and alignment of all principal numerical results.

**Location in revised manuscript:** Throughout the manuscript.

# Reviewer #4

## Major comments

**Comment:** Clarify the structure and granularity of the dataset and whether monthly data are available at facility level. Provide the monthly data underlying Figure 6.

**Response:** The revised Methods now state that the source data are monthly facility-level aggregate records. The final analytic dataset contains 32,178 facility-month records. Because individual identifiers were unavailable, the numerator is reported examination events rather than unique women. The national monthly series used for the interrupted time-series analysis is now provided as S3 Data in the Supporting Information.

**Location in revised manuscript:** Methods, Study design and setting and Data sources and reporting structure; Results, National reported CBE activity; S3 Data.

**Comment:** Reconsider the interpretation of partial-year 2025 data.

**Response:** This issue has been resolved by incorporating October through December 2025 program exports. The revised analysis now covers the complete 2025 calendar year. We recalculated all 2025 results and no longer annualize or extrapolate a partial-year numerator.

**Location in revised manuscript:** Methods; Results; Tables 1–5; Figs 1–6; Supporting Information.

**Comment:** Clarify and temper the interrupted time-series analysis, including level versus slope, seasonality, autocorrelation, missing reporting, changing completeness, and the short pre-intervention period.

**Response:** The revised Methods distinguish the immediate level-change and slope-change parameters and specify the segmented model. Calendar-month fixed effects address seasonality, and Newey-West standard errors with 12 monthly lags address serial correlation. Missing facility-month reports were not imputed. Sensitivity analyses use events per reporting facility, consistently reporting facilities, an April 2020 interruption point, and an autoregressive specification. Neither the immediate level change nor the slope change is statistically significant, and the Discussion explicitly notes the short pre-intervention period, changing reporting completeness, and observational nature of the analysis.

**Location in revised manuscript:** Methods, Data sources and reporting structure and Statistical analysis; Results, Interrupted time-series analysis; Discussion; Fig 6; Appendix Table A6.

**Comment:** Address low and potentially differential reporting completeness more fully and consider higher-completeness or consistent-reporting analyses.

**Response:** Reporting completeness is now explicitly defined and reported as a principal result. Annual completeness is presented for every study year, and 2025 variation is summarized across divisions and districts. An exploratory Spearman analysis assesses the association between district completeness and reported CBE examination rate. Fixed cohorts of facilities reporting in at least 9 months and at least 6 months of every year were added to test whether temporal patterns were driven by changes in the reporting set. The Discussion treats differential reporting as a potential source of geographic and temporal bias.

**Location in revised manuscript:** Methods, Outcomes and Statistical analysis; Results, Reporting completeness and Sensitivity analyses; Discussion; Appendix Tables A4 and A5.

**Comment:** Interpret CBE positivity cautiously and clarify whether the population is asymptomatic, symptomatic, or mixed. Where possible, report diagnostic completion and confirmed cancer.

**Response:** The routine dataset does not contain a reliable field distinguishing asymptomatic from symptomatic examinations, nor does it contain individual-level diagnostic completion or confirmed cancer outcomes. We therefore do not infer the clinical case mix. The revised manuscript explicitly states that participant symptoms, clinical indications, risk factors, referral completion, and diagnostic confirmation were unavailable. Positivity is defined only as an abnormal CBE finding among reported examinations, and we state that it cannot be interpreted as cancer detection or used to estimate positive predictive value or diagnostic sensitivity.

**Location in revised manuscript:** Methods, Outcomes; Results, CBE positivity; Discussion; limitations.

**Comment:** Strengthen interpretation of the NICRH linkage and clarify the denominator for the “untreated” estimate.

**Response:** We removed any implication of individual-level linkage. The registry is now presented as contextual data from a single tertiary cancer center. Table 6 labels the measure as “Patients untreated before presentation to NICRH” and provides the approximate numerator and denominator from the registry summary. The Discussion emphasizes that these patients cannot be assumed to represent individuals in the CBE dataset and that the CBE data cannot establish referral completion, treatment initiation, stage shift, or survival.

**Location in revised manuscript:** Methods; Results, Contextual NICRH registry indicators; Discussion; Table 6.

## Minor comments and supplementary materials

**Comment:** Avoid treating 2025 as a full year unless complete.

**Response:** The revised 2025 data are complete from January through December, so 2025 is now directly presented as a complete calendar year.

**Location in revised manuscript:** Methods; Results; Tables and figure captions.

**Comment:** Figure 6 should show observed values, fitted trends, the interruption point, and, if possible, uncertainty.

**Response:** Figure 6 now shows observed monthly reported CBE events, fitted model values, the counterfactual trajectory projected from the pre-intervention trend, and the March 2020 interruption point. Statistical uncertainty for the level and slope changes is reported numerically in the abstract, Results, and Appendix Table A6.

**Location in revised manuscript:** Fig 6; Abstract; Results; Appendix Table A6.

**Comment:** Clarify how missing months were handled.

**Response:** Missing facility-month reports were retained as missing reports and were not imputed. Reporting completeness is presented explicitly so that the resulting national monthly totals are interpreted as reported activity rather than complete national service volume.

**Location in revised manuscript:** Methods, Data sources and reporting structure and Outcomes; Discussion.

**Comment:** Revise wording around a sustained negative slope and separate descriptive findings from exploratory or limited findings.

**Response:** The revised manuscript no longer describes a statistically significant sustained negative slope. It states that neither the immediate level change nor the post-intervention slope change was statistically significant. The Discussion separates robust descriptive findings, such as reported event counts and completeness, from exploratory analyses and from interpretations limited by incomplete reporting.

**Location in revised manuscript:** Results, Interrupted time-series analysis; Discussion.

**Comment:** Expand the limitations to include event counts rather than unique women, differential reporting, ITS limitations, and inability to establish individual diagnostic and treatment pathways.

**Response:** These limitations are now stated explicitly. The revised limitations paragraph addresses incomplete and potentially differential reporting, lack of individual deduplication, approximate district denominators, missing demographics and clinical indications, absence of participant residence and private-sector utilization, absence of referral, diagnosis, stage, treatment, and survival data, the short pre-intervention period, temporal changes in reporting, and the contextual rather than linked nature of the NICRH registry data.

**Location in revised manuscript:** Discussion, limitations paragraph.

**Comment:** Add supplementary monthly data, completeness information, ITS details, sensitivity analyses, a data dictionary, and stratified analyses where available.

**Response:** We expanded the Supporting Information within the limits of the source data. S3 Data provides the monthly national series underlying Figure 6. Appendix Table A4 provides annual reporting completeness, Appendix Table A5 provides the consistent-reporting cohorts, and Appendix Table A6 provides interrupted time-series coefficients. Appendix Table A1 reports the denominator and high-volume-facility sensitivity analyses. Definitions of CBE positivity, reporting completeness, facility categories, missing reports, and the ITS variables are provided in the Methods and Supporting Information notes. A separate age-stratified analysis was not possible because age was not available at the individual level, and the rural versus urban stratification was removed because participant residence was unavailable. The suggested partial-year 2025 sensitivity analyses are no longer necessary because complete 2025 data were obtained.

**Location in revised manuscript:** Methods; S1 File, Appendix Tables A1, A4, A5, and A6; S3 Data.

**Comment:** Revise the title and abstract to avoid overstatement and make clear that the study uses aggregated routine program data.

**Response:** The revised title now begins with “Reported clinical breast examination activity” and no longer presents the study as national coverage or as a COVID-19-disruption analysis. The abstract states that the analysis uses routine program data, that records are CBE events rather than unique women, that reporting was incomplete, and that the results describe reported program activity rather than true population screening coverage. The interrupted time-series estimates and confidence intervals are reported with non-significant interpretation.

**Location in revised manuscript:** Title and Abstract.

# Reviewer #5

**Comment:** Review use of the term “screening” because routine CBE program data should generally be described as CBE examinations or receipt of CBE.

**Response:** We reviewed this terminology throughout. The Bangladesh routine-data results are now described as CBE examinations, reported CBE events, reported examination rates, and reported program activity. “Screening” is retained only where it refers to population screening concepts, mammographic screening, the titles or context of published screening studies, or statements explicitly explaining why the present estimates are not true screening coverage or evidence of screening effectiveness.

**Location in revised manuscript:** Throughout the manuscript, including Abstract, Methods, Results, Discussion, and captions.

**Comment:** Move the WHO Global Breast Cancer Initiative context into the Introduction and separate its early-stage diagnosis target from CBE activity.

**Response:** We added WHO Global Breast Cancer Initiative context to the Introduction immediately after the description of Bangladesh’s public CBE service expansion. The Discussion now clearly states that the initiative’s 60% target refers to invasive breast cancers diagnosed at stage I or II and is not a CBE examination-coverage target.

**Location in revised manuscript:** Introduction; Discussion, WHO Global Breast Cancer Initiative paragraph.

**Comment:** Provide more context on the Bangladesh CBE program and programmatic investment relative to WHO recommendations.

**Response:** We expanded the Introduction and study setting to describe public-sector service delivery through district hospitals and upazila health complexes, define the upazila level, describe monthly electronic reporting, and provide referral and tertiary breast-service context. The routine dataset does not contain validated measures of programmatic investment, awareness activities, training coverage, financing, or national diagnostic capacity. We therefore did not add unsupported quantitative claims about these components and limited the description to information that could be documented reliably.

**Location in revised manuscript:** Introduction; Methods, Study design and setting and Data sources and reporting structure.

**Comment:** High-income-country wording should recognize timely diagnosis as well as treatment.

**Response:** The Introduction now states that mortality reductions in higher-income settings reflect a combination of earlier diagnosis, organized screening in eligible populations, and access to effective treatment.

**Location in revised manuscript:** Introduction, opening paragraph.

**Comment:** Explain “upazila health complex.”

**Response:** The term is now defined in the Methods as a public-sector health facility serving a subdistrict administrative unit.

**Location in revised manuscript:** Methods, Study design and setting.

**Comment:** Add results on reporting completeness across time and geography.

**Response:** A dedicated Results subsection now reports annual completeness from 2019 through 2025, confirms that no study month reached 60% national completeness, reports 2025 division and district ranges, and provides an exploratory correlation between district completeness and reported CBE examination rate. Annual completeness is also provided in Appendix Table A4.

**Location in revised manuscript:** Results, Reporting completeness; Appendix Table A4.

**Comment:** Reference STROBE in the manuscript and include the checklist as Supporting Information.

**Response:** The Methods now state that reporting followed the STROBE recommendations, and the completed checklist is listed as S4 Checklist in the Supporting Information.

**Location in revised manuscript:** Methods, Statistical analysis; Supporting Information, S4 Checklist.

**Comment:** Clarify the limited role of the single tertiary-center registry in interpreting positivity and outcomes.

**Response:** The revised Methods and Discussion clearly state that NICRH is a single tertiary-center registry source and that it was not linked to the CBE data. We do not use the registry to infer diagnostic yield, treatment linkage, stage, or survival among people represented in the national CBE dataset. We also avoid claiming that low CBE positivity is explained by the NICRH registry.

**Location in revised manuscript:** Methods; Results, Contextual NICRH registry indicators; Discussion; Table 6.

**Comment:** The low radiology proportion and high proportion untreated before NICRH may need program context.

**Response:** We retained these registry indicators as descriptive context but avoided speculating about the availability or location of radiology, surgery, or other treatment services because the routine CBE dataset does not measure those system characteristics. The revised text explicitly limits the interpretation to patients who reached NICRH and does not generalize these registry measures to national CBE pathways.

**Location in revised manuscript:** Results, Contextual NICRH registry indicators; Discussion; Table 6.

**Comment:** There are many tables and figures and some could potentially be combined.

**Response:** We reviewed the presentation and removed the previous rural versus urban analysis. We retained the remaining six tables and six figures because each now addresses a distinct component requested during review, while detailed geographic and sensitivity material is placed in Supporting Information. This preserves transparency without expanding the main text with additional tables.

**Location in revised manuscript:** Tables 1–6; Figs 1–6; Supporting Information.

# Closing statement

We thank the Academic Editor and reviewers again for the detailed comments. The revision has materially changed the framing and analysis of the study. We believe the revised manuscript now more accurately reflects what can be supported by the aggregate routine program data, makes reporting completeness and missingness explicit, avoids unsupported population-level or causal interpretations, and provides the additional analyses and Supporting Information needed for transparent evaluation.
