# Supplementary material for: Reported clinical breast examination activity in Bangladesh, 2019–2025: Temporal trends, geographic variation, positivity, and reporting completeness
Source: PLoS One. 2026 Oct 29;21(10):e0361910. doi: 10.1371/journal.pone.0361910 (PMC13718965; doi:10.1371/journal.pone.0361910)
Supplement: Supplementary file 4 [file pone.0361910.s006.docx]

Response to the Academic Editor and Reviewers

**Manuscript ID:** PONE-D-26-23766R1

**Title:** Reported clinical breast examination activity in Bangladesh, 2019–2025: temporal trends, geographic variation, positivity, and reporting completeness

Dear Academic Editor and Reviewers,

Thank you for the careful second-round review of our manuscript. We appreciate that Reviewers 1, 2, and 4 considered their previous concerns addressed and that Reviewer 5 found the manuscript substantially improved. We have now addressed all remaining points raised by Reviewer 3 and the minor editorial suggestions from Reviewer 5. We also repeated the relevant data and statistical audits before making any textual changes.

The principal changes in this revision are:

- Re-audit of all district denominator assignments against the Bangladesh Population and Housing Census 2022 source, correction of six misassigned district denominator series, and recalculation of district rates, rankings, Table 3, Fig 3, the district-level correlation, and coded district-level Supporting Information.
- Reconciliation of the 32,178 facility-month records with the reporting-completeness series by using location-specific facility identity (district plus normalized facility name), correcting the eligible facility roster and annual submitted-report counts.
- Expansion of the fixed-facility-cohort sensitivity table to include annual submitted facility-month reports and CBE events per submitted report.
- Expansion of the interrupted time-series supplement to report the April 2020 interruption and autoregressive-error sensitivity models, and revision of S3 so that it contains observed monthly data and model-design variables rather than unconstrained fitted/counterfactual predictions.
- Revision of the geographic-data description from “anonymized” to “coded,” with explicit acknowledgement that some codes may be inferable from published aggregate values.
- The three minor wording changes suggested by Reviewer 5.

# Response to the Academic Editor

**Comment:** The manuscript has been substantially improved, and most reviewers are satisfied with the revisions. Before acceptance, the authors are required to address the remaining concerns raised by Reviewer #3, particularly regarding data accuracy, consistency, and statistical reporting, and correct any identified errors. The minor editorial suggestions from Reviewer #5 are also recommended.

**Response:** We agree and have completed a targeted re-audit of the underlying district denominators, facility identifiers, reporting-completeness calculations, fixed-cohort reporting patterns, and interrupted time-series sensitivity analyses. The identified errors were corrected in the manuscript and Supporting Information, and all downstream quantities affected by those corrections were regenerated. We did not alter analyses or text that were not implicated by the remaining reviewer comments. The minor wording changes requested by Reviewer 5 were also made.

# Response to Journal Requirements

**Comment:** Please evaluate any reviewer-suggested citations for relevance, and review the reference list for completeness, correctness, and retracted publications.

**Response:** No new citations were requested by the reviewers in this round. The reference list was reviewed in conjunction with the targeted revisions, and no reference changes were required for the issues addressed in this revision.

# Reviewer #1

**Comment:** All comments have been addressed.

**Response:** Thank you for confirming that the previous comments were addressed. No additional changes were required in response to Reviewer 1.

# Reviewer #2

**Comment:** All comments have been addressed. The revised version has sufficient clarity and readability.

**Response:** Thank you for the positive assessment. No additional changes were required in response to Reviewer 2.

# Reviewer #3

**Comment:** 1. Please verify the district denominators. Table 3 assigns 609,718 eligible women to Cumilla and 1,454,114 to Cox’s Bazar in 2025. This appears inconsistent with the stated proportional allocation from the 2022 census, which records a substantially larger female population in Cumilla than in Cox’s Bazar. Please check how district census populations were matched to examination counts across all years and update the geographic rates, rankings, and supplementary data if needed.

**Response:** Thank you for identifying this problem. We re-audited the denominator assignment for all 64 districts across all seven study years against the Bangladesh Population and Housing Census 2022 source. We confirmed a district-alignment error affecting six district denominator series: Cumilla and Cox’s Bazar were interchanged, and four districts within Rajshahi division had a within-division denominator permutation. We corrected those assignments for every study year and regenerated all district-level rates, rankings, Table 3, Fig 3, the 2025 district completeness-rate correlation, and S2 Data. Because the misassignments occurred within the same divisions, national and division-level denominators and rates were unchanged. After correction, the district rate range was <0.1% in Sunamganj to 1.2% in Joypurhat in 2019; 0.8% in Sunamganj to 6.8% in Shariatpur in 2023; and 0.2% in Kurigram to 2.8% in Rajshahi in 2025. The exploratory 2025 district-level correlation is now Spearman ρ = −0.151, P=0.235.

**Location in revised submission:** Results, Geographic variation and Reporting completeness; Table 3; Fig 3; S2 Data; related Supporting Information descriptions.

**Comment:** 2. Please reconcile the facility-month count. The manuscript reports 32,178 facility-month records, whereas the annual submitted-report counts in S1 Table A4 sum to 32,044, matching the monthly counts in S3. If these figures refer to different types of records, please explain the distinction.

**Response:** We reconciled this discrepancy and confirmed that 32,178 is the correct number of submitted location-specific facility-month records. The previous completeness calculation identified facilities by normalized facility name alone. Three facility names were shared by distinct facilities in different districts; when same-named facilities reported in the same month, the name-only identifier collapsed separate records. This accounted for exactly 134 missing facility-month reports in the earlier completeness series (32,178 − 32,044). We now identify facilities by district plus normalized facility name. This correction changes the baseline location-specific roster from 1,206 to 1,209 facilities and the late-2025 sensitivity roster from 1,213 to 1,216. Annual submitted-report counts and reporting-completeness estimates were recalculated in S1 Table A4 and S3. The substantive interpretation is unchanged: reporting remained incomplete, and no month reached 60% national completeness.

Location in revised submission: Abstract; Methods; Methods, Study design and setting, Data sources and reporting structure, Outcomes, and Statistical analysis; Results, Reporting completeness; Discussion; S1 Table A4; S3 Data.

**Comment:** 3. Please add reporting counts for the fixed facility cohorts. S1 Table A5 shows declining examination counts after 2023. The number of submitted facility-month reports, or examinations per submitted report, for each cohort and year would help readers assess how much changing reporting frequency contributed to that decline.

**Response:** We agree. S1 Table A5 now reports, for each year, the number of submitted facility-month reports and CBE events per submitted report for both fixed cohorts in addition to the original examination-event totals. From 2023 to 2025, submitted reports changed only from 572 to 570 in the strict 48-facility cohort, while CBE events per submitted report decreased from 212.4 to 127.0. In the broader 118-facility cohort, submitted reports changed from 1,408 to 1,382, while events per submitted report decreased from 184.8 to 113.9. We added these results to the manuscript. These findings indicate that the post-2023 decline within the fixed cohorts was not primarily explained by reduced reporting frequency.

**Location in revised submission:** Results, Sensitivity analyses; S1 Table A5.

**Comment:** 4. Please complete the interrupted time-series results in the supplement. The manuscript mentions analyses using an April 2020 interruption and autoregressive errors, but their estimates are not shown in S1 Table A6. Please report these results or qualify the statement about their agreement with the primary analysis. Please also check the fitted series in S3, which gives a negative fitted examination count for April 2019.

Response: We have expanded S1 Table A6 to report the full parameter estimates, 95% confidence intervals, and P values for both the April 2020 interruption sensitivity model and the autoregressive-error sensitivity model. Both remain consistent with the primary conclusion of no statistically significant immediate level change or post-intervention slope change. For the April 2020 model, the immediate level change was 17,120 events (95% CI −3,821 to 38,060; P=0.109) and the slope change was −233 events/month (95% CI −1,468 to 1,002; P=0.712). For the AR(1)-error model, the immediate level change was 14,416 events (95% CI −6,650 to 35,482; P=0.180) and the slope change was −375 events/month (95% CI −10,964 to 10,214; P=0.945). We also checked the April 2019 value in the former S3 fitted series. The observed count was positive (10,434); the negative value was an unconstrained fitted prediction from the linear segmented model rather than an observed count. To avoid presenting model predictions as underlying data, S3 now provides the observed monthly CBE series, reporting-facility counts, reporting completeness, events per reporting facility, and the model-design variables used in the ITS analyses. Model estimates are reported in S1 Table A6. The Fig 6 caption now also clarifies that the segmented linear model is unconstrained and that fitted values may fall below zero in low-volume months; these are model predictions rather than observed counts. The primary model specification and inference were not changed.

Location in revised submission: S1 Table A6; S3 Data; Fig 6 caption; Supporting Information description in the main manuscript.

**Comment:** 5. Please clarify the description of the geographic codes. Although S2 omits district names, exact counts in Table 3 allow some codes to be matched to named districts. Please describe the data as coded rather than fully anonymized and ensure that this presentation meets the journal’s request concerning location data.

**Response:** We agree. We no longer describe S2 as anonymized. It is now labeled “coded district-level” data. District names and the code key are not included in S2, but the accompanying Read Me and manuscript Supporting Information description explicitly state that some district codes may be inferable by comparison with published aggregate values. The dataset contains only aggregate district-year data and no individual-level identifiers. This wording more accurately describes the privacy properties of the file while retaining the location-data protections requested by the journal.

**Location in revised submission:** S2 Data; S1 geographic-code note; Supporting Information captions/descriptions in the main manuscript.

# Reviewer #4

**Comment:** The authors have satisfactorily addressed the substantive comments from the previous round, and no further substantive methodological concerns remain.

**Response:** Thank you for confirming that the previous substantive concerns were addressed. No additional changes were required in response to Reviewer 4.

# Reviewer #5

**Comment:** Please remove or briefly revise the sentence stating that the previous characterization of facility-level differences as rural-versus-urban population inequity was not supported and had been replaced by facility-type analysis.

**Response:** We removed the self-referential sentence describing how the previous version had characterized rural-versus-urban inequity. The surrounding discussion now directly states the current interpretation without referring to the revision history.

**Location in revised submission:** Discussion, Geographic variation paragraph.

**Comment:** Please remove or briefly revise the sentence beginning “The revised analysis includes the complete 2025 calendar year…”

**Response:** We revised this sentence to remove the self-referential phrase “The revised analysis.” It now begins “The analysis includes…” while retaining the substantive description of the study’s strengths.

**Location in revised submission:** Discussion, Strengths paragraph.

**Comment:** Very minor: “Reporting completeness remained incomplete throughout the study period” contains duplicate wording; remove the word completeness.

**Response:** Corrected as suggested. The sentence now reads: “Reporting remained incomplete throughout the study period.”

**Location in revised submission:** Results, Reporting completeness.

# Closing

We thank the Academic Editor and reviewers again for their careful review. We believe these targeted corrections resolve the remaining concerns regarding data accuracy, internal consistency, statistical reporting, and geographic-data description while preserving the analyses and presentation that were already considered satisfactory.
