# Supplementary material for: Reported clinical breast examination activity in Bangladesh, 2019–2025: Temporal trends, geographic variation, positivity, and reporting completeness
Source: PLoS One. 2026 Oct 29;21(10):e0361910. doi: 10.1371/journal.pone.0361910 (PMC13718965; doi:10.1371/journal.pone.0361910)
Supplement: Peer review history

# Peer review: Decision Letter 0

**DOI:** 10.1371/journal.pone.0361910.r001

**Authors:** Qi Zhou

10 Jul 2026

Dear Dr. Das,

Thank you for submitting your manuscript to PLOS ONE. After careful consideration, we feel that it has merit but does not fully meet PLOS ONE’s publication criteria as it currently stands. Therefore, we invite you to submit a revised version of the manuscript that addresses the points raised during the review process.

==============================

The reviewers recognize the relevance of this topic and the potential value of this large routine program dataset for understanding clinical breast examination activity in Bangladesh. I agree that the study addresses an important public health issue and could make a useful contribution after appropriate revision.

However, the manuscript requires major revision before it can be considered further. The reviewers raised important concerns regarding methodological clarity, reporting completeness, interpretation of aggregate routine data, terminology, statistical analysis, presentation of partial-year data, and the extent to which the conclusions are supported by the available evidence. These concerns should be addressed carefully in the revised manuscript and in a detailed point-by-point response.

I do not see substantive conflicts among the reviewer comments. The reviewers differ mainly in emphasis, and the authors should consider all comments constructively. Where the data allow, additional clarification or analysis should be provided; where the data are limited, the manuscript should acknowledge those limitations clearly and temper the conclusions accordingly.

I encourage the authors to revise the manuscript thoroughly. With improved methodological transparency, clearer terminology, careful attention to the reviewers’ comments, and more cautious interpretation of the findings, the manuscript may become a stronger and more useful contribution to the literature.

==============================

Please submit your revised manuscript by Sep 08 2026 11:59PM. If you will need more time than this to complete your revisions, please reply to this message or contact the journal office at plosone@plos.org. When you're ready to submit your revision, log on to https://www.editorialmanager.com/pone/ and select the 'Submissions Needing Revision' folder to locate your manuscript file.

- A letter that responds to each point raised by the academic editor and reviewer(s). You should upload this letter as a separate file labeled 'Response to Reviewers'.
- A marked-up copy of your manuscript that highlights changes made to the original version. You should upload this as a separate file labeled 'Revised Manuscript with Track Changes'.
- An unmarked version of your revised paper without tracked changes. You should upload this as a separate file labeled 'Manuscript'.

If you would like to make changes to your financial disclosure, please include your updated statement in your cover letter. Guidelines for resubmitting your figure files are available below the reviewer comments at the end of this letter.

If applicable, we recommend that you deposit your laboratory protocols in protocols.io to enhance the reproducibility of your results. Protocols.io assigns your protocol its own identifier (DOI) so that it can be cited independently in the future. For instructions see: https://journals.plos.org/plosone/s/submission-guidelines#loc-laboratory-protocols. Additionally, PLOS ONE offers an option for publishing peer-reviewed Lab Protocol articles, which describe protocols hosted on protocols.io. Read more information on sharing protocols at https://plos.org/protocols?utm_medium=editorial-email&utm_source=authorletters&utm_campaign=protocols.

As the corresponding author, your ORCID iD is verified in the submission system and will appear in the published article. PLOS supports the use of ORCID, and we encourage all coauthors to register for an ORCID iD and use it as well. Please encourage your coauthors to verify their ORCID iD within the submission system before final acceptance, as unverified ORCID iDs will not appear in the published article. *Only*  the individual author can complete the verification step; PLOS staff *cannot*  verify ORCID iDs on behalf of authors.

We look forward to receiving your revised manuscript.

Kind regards,

Qi Zhou

Guest Editor

PLOS One

**Journal Requirements:**

When submitting your revision, we need you to address these additional requirements.

1. Please ensure that your manuscript meets PLOS ONE's style requirements, including those for file naming. The PLOS ONE style templates can be found at

https://journals.plos.org/plosone/s/file?id=wjVg/PLOSOne_formatting_sample_main_body.pdf and

https://journals.plos.org/plosone/s/file?id=ba62/PLOSOne_formatting_sample_title_authors_affiliations.pdf

2. Please note that your Data Availability Statement is currently missing the DOI/accession number of each dataset OR a direct link to access each database. If your manuscript is accepted for publication, you will be asked to provide these details on a very short timeline. We therefore suggest that you provide this information now, though we will not hold up the peer review process if you are unable.

3. Your ethics statement should only appear in the Methods section of your manuscript. If your ethics statement is written in any section besides the Methods, please delete it from any other section.

4. Please include captions for your Supporting Information files at the end of your manuscript, and update any in-text citations to match accordingly. Please see our Supporting Information guidelines for more information: http://journals.plos.org/plosone/s/supporting-information.

5. We note that there is identifying data in the Supporting Information file < Appendix_A2_district_full.csv and Supplementary_Appendix_CBE_Bangladesh.docx >. Due to the inclusion of these potentially identifying data, we have removed this file from your file inventory. Prior to sharing human research participant data, authors should consult with an ethics committee to ensure data are shared in accordance with participant consent and all applicable local laws.

Data sharing should never compromise participant privacy. It is therefore not appropriate to publicly share personally identifiable data on human research participants. The following are examples of data that should not be shared:

-Name, initials, physical address

-Ages more specific than whole numbers

-Internet protocol (IP) address

-Specific dates (birth dates, death dates, examination dates, etc.)

-Contact information such as phone number or email address

-Location data

-ID numbers that seem specific (long numbers, include initials, titled “Hospital ID”) rather than random (small numbers in numerical order)

Data that are not directly identifying may also be inappropriate to share, as in combination they can become identifying. For example, data collected from a small group of participants, vulnerable populations, or private groups should not be shared if they involve indirect identifiers (such as sex, ethnicity, location, etc.) that may risk the identification of study participants.

Additional guidance on preparing raw data for publication can be found in our Data Policy (https://journals.plos.org/plosone/s/data-availability#loc-human-research-participant-data-and-other-sensitive-data) and in the following article: http://www.bmj.com/content/340/bmj.c181.long.

Please remove or anonymize all personal information ( location data in column B of Appendix_A2_district_full.csv. and location data in Appendix Table A3 ), ensure that the data shared are in accordance with participant consent, and re-upload a fully anonymized data set. Please note that spreadsheet columns with personal information must be removed and not hidden as all hidden columns will appear in the published file.

6. If the reviewer comments include a recommendation to cite specific previously published works, please review and evaluate these publications to determine whether they are relevant and should be cited. There is no requirement to cite these works unless the editor has indicated otherwise.

**Additional Editor Comments:**

Thank you for submitting this important study. The reviewers recognize the value of the large national dataset and the relevance of the topic. However, substantial revision is required before the manuscript can be considered further. The main issues concern low reporting completeness, uncertainty about whether the data represent unique women or examinations, the interpretation of 2025 partial-year data, the rural–urban comparison, the interrupted time-series analysis, and the use of terms such as “screening” and “national coverage.” Please address all reviewer comments carefully, provide additional analyses where feasible, revise the terminology and conclusions accordingly, and ensure full numerical and methodological consistency throughout the manuscript.

[Note: HTML markup is below. Please do not edit.]

Reviewers' comments:

Reviewer's Responses to Questions

**Comments to the Author**

1. Is the manuscript technically sound, and do the data support the conclusions?

Reviewer #1: Partly

Reviewer #2: Partly

Reviewer #3: No

Reviewer #4: Partly

Reviewer #5: Yes

**********

2. Has the statistical analysis been performed appropriately and rigorously?

Reviewer #1: Yes

Reviewer #2: No

Reviewer #3: No

Reviewer #4: No

Reviewer #5: Yes

**********

3. Have the authors made all data underlying the findings in their manuscript fully available?

The PLOS Data policy

Reviewer #1: Yes

Reviewer #2: No

Reviewer #3: No

Reviewer #4: No

Reviewer #5: Yes

**********

4. Is the manuscript presented in an intelligible fashion and written in standard English?

Reviewer #1: Yes

Reviewer #2: Yes

Reviewer #3: Yes

Reviewer #4: Yes

Reviewer #5: Yes

**********

Reviewer #1: Major Concerns

1. Fundamental Data Quality Issues Undermine the Analysis

The authors report that "reporting completeness was suboptimal, averaging only 31% nationally." This is a critical methodological flaw that is insufficiently addressed:

How can coverage estimates be reliable when ~69% of facilities did not report? The manuscript presents coverage as actual population coverage (e.g., 2.4% in 2023), but this likely represents reporting facility coverage rather than true national coverage.

The sensitivity analysis restricting to months with ≥60% reporting completeness "yielded no valid observations, reflecting systemic under-reporting." This should have been a red flag to reconsider the primary analytical approach.

The ITS analysis showing a post-lockdown decline of -4,699 examinations per month could simply reflect declining reporting completeness rather than true service disruption.

Recommendation: The authors must either:

Develop and validate imputation methods for missing facility data, or

Reframe the analysis as "reported coverage among reporting facilities" and clearly acknowledge the severe data limitations in the title and abstract, OR

Exclude years with <60% reporting completeness and conduct a more robust analysis of the high-quality years only

2. Denominator Calculation Is Problematic

District-level denominators were proportionally allocated from census totals without age-specific distributions. This is acknowledged but the impact is substantial—the authors are effectively assuming uniform age distribution across districts, which is unlikely to hold.

Using women aged 30-69 years includes the full eligible population, but facility-level screening counts include all women who presented for any reason, not population-based active recruitment. This denominator mismatch makes the "coverage" metric questionable—it's more accurately described as utilization rate rather than population screening coverage.

The alternative denominator of 30-64 years changes coverage from 1.3% to 1.4%—a 7.7% relative difference that deserves more discussion, not just a brief sensitivity mention.

3. Counterintuitive Findings Lack Adequate Explanation

Rural coverage consistently exceeding urban coverage (1.9% vs 0.2% in 2023) is counterintuitive given typical urban healthcare advantage. The authors attribute this to "more effective deployment in upazila-level facilities than in urban tertiary centers," but this requires:

Data on number and distribution of facilities—are there more rural facilities?

Evidence on resource allocation—is CBE service delivery actually prioritized in rural areas, or is the difference a denominator artifact?

Urban denominator issues—urban populations include wealthier women who may access private screening not captured in public program data, artificially lowering measured urban coverage.

4. Positivity Rate Interpretation Requires Caution

The mean positivity rate of 1.0% is substantially lower than the 3.7% reported in other LMIC CBE programs (Mittra et al., BMJ 2021; Pace et al., BMJ Glob Health 2025). The authors attribute this to "differences in screening quality, patient selection, or reporting practices," but this is speculative without:

Quality assurance data on examiner training and performance

Patient demographic data (age distribution, risk factors)

Data on referral completion and diagnostic confirmation rates

False-positive rates—without complete follow-up, the clinical utility of CBE positivity is unknown

5. Incomplete Linkage to Outcomes and Survival Data

Only 80.5% of cancers had histopathological confirmation—this is good but the missing 19.5% (310 cases) were diagnosed by cytology (15.3%) or radiology/clinical alone (4.0%), which are less reliable for cancer diagnosis.

"More than three-quarters of women were untreated before reaching NICRH" is a striking finding, but:

This is based on registry data from 2018-2020, while the screening data are from 2019-2025—are these populations comparable?

No survival or stage data are provided, which is the ultimate measure of screening effectiveness.

The manuscript promises "linkage to cancer registry data for contextualization" but the linkage is descriptive rather than integrated—there's no analysis matching screened women to cancer outcomes.

Minor Concerns

1. Methods Section Needs Clarification

"Facility reporting completeness varied by month"—how was completeness defined? Percentage of facilities reporting? Percentage of expected reports received?

"Data were curated and cleaned"—what specific procedures were used? How many duplicate rows, mismatched codes, or implausible values were removed? This is critical for reproducibility.

The ITS analysis specification is incomplete—were seasonality or autocorrelation accounted for? What was the model structure? Were covariates included?

2. Results Presentation Needs Improvement

Tables and figures often duplicate information without adding value

Figure 1 shows coverage trends but does not show monthly counts underlying the ITS analysis

The rural-urban gap (Table 4) uses national denominators for both rural and urban coverage, which is unconventional. Standard approach would use rural/urban-specific population denominators. This requires justification or recalculation.

3. Discussion of Global Benchmarks Is Misleading

The authors compare Bangladesh coverage (0.3-2.4%) to the WHO GBCI target of 60% early diagnosis coverage by 2040. This comparison is not appropriate—Bangladesh's CBE coverage is a screening coverage measure, while the 60% target refers to stage at diagnosis (proportion of cancers diagnosed at stage I or II). These are conceptually different metrics.

4. Reference List Quality

Several references are to websites accessed at future dates (e.g., "Accessed November 24, 2025")—this manuscript was submitted before these access dates. This suggests potential backdating of citations or manuscript preparation timeline issues. PLOS ONE policy requires actual access dates.

Reference 64 (Raghavan and Jatoi, 2024) is cited in the conclusion to emphasize "improving cancer treatment infrastructure," but this article actually argues against prioritizing mammography in developing countries. The citation seems misused.

5. Policy Recommendations Are Generic and Not Derived from Findings

The call for "updated policy frameworks, investment in screening, and registry integration" is not novel and does not flow directly from the data presented.

The authors list examples of successful registries in India, Nepal, Iran, Malaysia, Kenya, Uganda, and Rwanda, but provide no specific lessons relevant to Bangladesh's context.

No concrete implementation recommendations are provided (e.g., specific DHIS2 integration steps, training requirements, financing mechanisms).

Reviewer #2: Dear Authors,

Thank you very much for your manuscript. Although the topic is not new in the literature, your work offers interesting insights, as it describes the experience of Bangladesh, a setting that remains underrepresented in the scientific literature, thereby contributing to the available evidence on this issue.

However, several revisions, both major and minor, are necessary and are listed below.

Title: The term “COVID-19” could be removed to improve the clarity and readability of the title.

Short title: The short title should be clearer. In particular, the acronym “CBE” could be replaced.

Correspondence to: Private email addresses should be avoided whenever possible. Therefore, if available, the corresponding author should provide an institutional email address.

Introduction: Overall, the Introduction should provide more details regarding the recommended mammographic screening program in Bangladesh. Is mammographic screening also recommended in this setting? Is it currently implemented? If so, what is the level of adherence? Further details should also be provided regarding patient management. Are national recommendations available? Are there referral centers? Are breast units available?

Page 3, line 58: A plural form may be more appropriate.

The term “programme,” which appears in some parts of the manuscript, should be standardized to “program.”

Overall, the Methods section should provide more details on whether data were reported by peripheral centers through a secure platform, electronic spreadsheets, or paper forms, and how frequently data were collected. This would help clarify potential reporting-related biases. In addition, the authors should specify which types of information were collected locally.

The term “upazila” should be better explained, as not all readers may be familiar with it.

With regard to the marked decrease in assessments observed in 2023, the possible causes and any concomitant events should be more clearly described to improve interpretation of the results.

Finally, the differences in participation between rural and urban settings should be better discussed. Could this difference represent a positive rather than a negative finding, possibly reflecting participation in mammography-based screening programs in urban areas?

A definition of NICRH should be provided at first mention.

In the tables, the use of commas to indicate thousands is recommended to facilitate readability. In addition, centering the content of the cells would improve clarity.

Finally, a statistical revision is suggested to improve it with more than descriptive analyses trying to focus on possible tests of association.

Reviewer #3: This manuscript addresses an important and under-researched public-health issue. National evidence on breast-cancer early detection in Bangladesh remains limited, and the use of routine programme data spanning more than six years and covering 1,206 facilities is a meaningful strength. The large volume of recorded CBE activity, the geographic analyses, the assessment of positivity, and the attempt to evaluate reporting completeness add value. The topic is highly relevant to cancer-control policy, programme monitoring, and health-system planning in Bangladesh.

The main limitation is the low reporting completeness. The manuscript reports an average completeness of 31%, and no month met the authors’ 60% threshold. The results therefore reflect reported activity from incompletely reporting facilities rather than complete national coverage. This affects temporal trends, geographic comparisons, positivity estimates, and the interrupted time-series analysis. Please use terms such as “reported CBE coverage” or “reported programme activity,” present completeness by year and geographic area, and, where possible, analyse facilities that reported consistently over time.

It is unclear whether the numerator represents unique women or examinations. The data are aggregate facility-month counts, and individual identifiers were unavailable. Repeat attendance across months, years, or facilities may therefore not have been identifiable. Please clarify what the numerator represents and, unless deduplication occurred before aggregation, describe the total as CBE examinations, attendances, or screening events rather than unique women.

The rural–urban analysis may be better interpreted as a comparison of reported CBE activity by facility location rather than coverage among rural versus urban women, because the classification is based on the facility rather than the patient’s residence and separate rural and urban population denominators were not used. In Table 4, both groups are divided by the same national denominator, and the gap signs also appear inconsistent; for example, 1.9% minus 0.2% would equal +1.7 percentage points. Please consider relabelling the analysis accordingly and revising the gap calculation.

The 2025 findings are not directly comparable with complete calendar years because they cover only January through September. Please compare January–September 2025 with the same months in previous years, report monthly rates, or otherwise account for the shorter period. Please do not interpret the 2025 value as a full-year decline without a comparable analysis.

The interrupted time-series analysis is insufficiently described. The estimated slope change of −4,699 examinations per month has a 95% confidence interval of −9,721 to +322, which includes zero, while the immediate level change was positive. Please provide the full model specification and coefficient table and avoid presenting the findings as definitive evidence of a sustained or causal COVID-19 effect.

Several numerical inconsistencies also require correction. The comparison between 3.1% and 1.5% is about two-fold, not more than six-fold. Sunamganj coverage was 0.8% in 2023, not below 0.1%. Table 4 reports negative differences while the text describes a rural advantage. Excluding the highest-volume 5% of facilities reduces mean coverage from 1.3% to 0.9%, which is a meaningful change. Please perform a full consistency check across the abstract, text, tables, figures, and supplementary material.

Finally, please revise the manuscript throughout for clarity, grammar, terminology, and numerical consistency.

Reviewer #4: Summary of strengths, weaknesses, and overall contribution

This is an interesting and potentially relevant study on clinical breast examination (CBE) coverage among the target population in Bangladesh, using secondary data from a national program database from 2019 to September 2025. Breast cancer is a highly prevalent condition and a major public health issue in Bangladesh, and the manuscript addresses an important evidence gap by attempting to describe, possibly for the first time at this scale, national patterns of breast cancer early detection through CBE.

The main strength of the article is its public health relevance. The study uses a large routine dataset, includes national and subnational coverage estimates, explores geographic and urban–rural differences, examines positivity rates, and attempts to assess the disruption associated with the COVID-19 lockdown using interrupted time-series analysis. These are all potentially valuable contributions for policy, planning, and health system strengthening.

However, the manuscript requires major revision before it can be considered for publication. The main concerns relate to methodological clarity, interpretation of results, and the way data are presented in tables and figures. The study appears to rely on aggregated secondary data rather than individual-level data, at least as currently reported. This has important implications for the interpretation of coverage, positivity rates, equity gaps, and linkage to care. In addition, some conclusions, particularly regarding the decline in 2025 and the post-pandemic trend, seem stronger than what the data and statistical uncertainty currently support.

Overall, the article addresses a relevant topic and may make an important contribution after substantial revision. However, the authors need to better clarify the data structure, strengthen the methodological description, revise the interpretation of incomplete 2025 data, and align the conclusions more closely with the limitations of routine aggregated data.

Major comments

Clarify the structure and granularity of the dataset

The manuscript should provide a clearer description of the dataset used for analysis. The article states that routine program data were used, but it is not sufficiently clear whether the numerator represents unique women screened or total examinations performed. This distinction is essential for interpreting coverage. If repeat examinations are possible and the data count examinations rather than individuals, coverage may be overestimated at the individual level.

In the dataset presented, the available data appear to be aggregated by region and year. However, Figure 6 presents monthly aggregated data for the whole country. This creates uncertainty regarding the actual granularity of the data used in the analysis. The authors should clarify whether monthly data were available for all facilities, districts, and years, or only at the national level. The monthly dataset underlying Figure 6 should be made available as supplementary material.

Reconsider the interpretation of 2025 data

A major concern is the treatment and interpretation of 2025. The manuscript reports data only from January to September 2025. Although the authors state that 2025 denominators were extrapolated proportionally, the numerator is also based on incomplete program data. This approach assumes that screening activity and reporting completeness are reasonably uniform across the calendar year, which may not be true for routine health service data.

The authors should avoid presenting 2025 as directly comparable with complete calendar years unless they provide additional justification or sensitivity analysis. A more appropriate approach would be to either incorporate complete 2025 data, compare January–September periods across all years, or present 2025 explicitly as a partial-year estimate. The conclusion that coverage or positivity declined in 2025 should be softened unless this finding is supported by a comparable analytic strategy.

This issue is particularly important because Figure 6 appears to suggest some seasonal variation, including increases in CBE volumes toward the end of some years. If this pattern is real, using January–September 2025 to infer an annual decline may be misleading.

Clarify and temper the interrupted time-series interpretation

The use of interrupted time-series analysis is appropriate in principle, given the clearly defined COVID-19 lockdown and the availability of repeated program data. However, the manuscript needs to provide more detail on the model specification.

The reported sustained negative slope of –4,699 examinations per month has a 95% confidence interval ranging from –9,721 to 322. Since this interval includes zero, the evidence for a statistically significant post-lockdown negative slope is not conclusive at the conventional 5% level. The manuscript should therefore avoid overly strong wording suggesting a definitive sustained decline unless additional analyses support this interpretation.

The authors should clarify whether the coefficient represents an immediate level change, a change in slope, or both. They should also report how autocorrelation, seasonality, missing reporting months, and changes in reporting completeness over time were handled. The relatively short pre-intervention period should also be acknowledged as a limitation of the interrupted time-series analysis.

Address low reporting completeness more fully

The reported national reporting completeness of approximately 31% is a major limitation. The current manuscript acknowledges this issue, but the implications should be more fully integrated into the interpretation.

Low reporting completeness does not only mean that true screening coverage may be higher than recorded. It may also introduce differential bias across districts, divisions, facility types, and urban–rural settings. Facilities or regions with better organization may report more completely, which could make apparent coverage differences reflect reporting quality rather than true service delivery. Therefore, the authors should be cautious when interpreting geographic and urban–rural differences as equity gaps.

The manuscript would be strengthened by presenting reporting completeness by year, division, district, and facility type. If possible, sensitivity analyses restricted to areas or facilities with higher reporting completeness would help assess the robustness of the findings.

Interpret CBE positivity rates with caution

The manuscript provides a clear operational definition of a positive screening test as any abnormal breast finding, including palpable lump, skin changes, nipple retraction, axillary lymphadenopathy, or other suspicious abnormalities. This is helpful. However, the interpretation of positivity rates still requires caution.

A positive CBE represents a clinical abnormality requiring further assessment, not a confirmed cancer diagnosis. The authors should clarify whether women undergoing CBE were asymptomatic participants in a screening program, symptomatic women seeking care, or a mixture of both. This distinction is important because positivity rates may reflect underlying case mix, referral patterns, provider training, and diagnostic suspicion rather than true screening yield.

Where possible, the manuscript should report the proportion of CBE-positive women who completed diagnostic assessment and the proportion with confirmed breast cancer.

Strengthen the discussion of linkage to diagnosis and treatment

The manuscript reports that more than three-quarters of women were untreated before reaching NICRH. This is an important finding, but it should be interpreted cautiously. The linkage appears to rely on a single cancer center registry, which may not represent the national pathway of all women with positive CBE or confirmed breast cancer.

The authors should clarify the denominator for this estimate: all women with positive CBE, all women diagnosed with breast cancer, or only women who reached NICRH. They should also specify how “untreated” was defined. If this estimate is based only on women arriving at NICRH, the conclusion should not be generalized to national treatment linkage without strong caveats.

Minor comments

The authors should avoid interpreting 2025 as a full year unless the data are complete or the analysis explicitly accounts for the partial-year nature of the data.

Figure 6 should include sufficient information to interpret the interrupted time-series analysis, including observed values, fitted trends, the lockdown point, and, if possible, confidence intervals around the counterfactual and post-intervention trends.

The manuscript should report whether missing months were treated as zero, excluded, imputed, or otherwise handled.

The wording around “sustained negative slope” should be revised to reflect the statistical uncertainty of the estimate.

The discussion should more explicitly separate findings that are descriptive and robust from those that are exploratory or limited by incomplete data.

Limitations

The limitations section already acknowledges several important issues, including estimated district-level denominators, suboptimal reporting completeness, limited linkage to a single cancer center registry, missing clinical metadata, and uncertainty in interpreting positivity rates. However, additional limitations should be explicitly included.

First, the partial nature of 2025 data should be acknowledged and should directly affect the interpretation of the reported decline. Second, the authors should clarify whether the analysis counts unique women or total examinations. Third, the possibility of differential reporting completeness should be discussed as a source of bias in geographic and urban–rural comparisons. Fourth, limitations of the interrupted time-series analysis should be addressed, including possible autocorrelation, seasonality, missing months, changes in reporting completeness, and the relatively short pre-intervention period. Finally, the manuscript should acknowledge that aggregated routine data cannot fully establish individual-level screening pathways, diagnostic confirmation, treatment initiation, or loss to follow-up.

Supplementary Materials

The manuscript would benefit from additional supplementary materials, including:

The monthly national dataset underlying Figure 6.

Annual and monthly numbers of CBE examinations, denominators, and coverage estimates.

Reporting completeness by year, division, district, and facility type.

A detailed description of the interrupted time-series model, including variables, assumptions, diagnostics, and handling of autocorrelation and seasonality.

Sensitivity analyses excluding 2025, annualizing 2025 with caveats, or comparing January–September periods across all years.

A data dictionary defining CBE positivity, reporting completeness, facility reporting status, and treatment linkage variables.

If available, stratified analyses by age group, facility type, and urban–rural classification.

Title and abstract

The title appears broadly appropriate if it accurately reflects that the study analyzes routine program data on CBE coverage rather than individual-level screening effectiveness. However, the abstract should be revised to avoid overstatement.

In particular, the statement that coverage declined in 2025 should specify that 2025 includes January to September only and is based on a partial-year denominator. The interrupted time-series result should also be presented more cautiously, given that the confidence interval for the post-lockdown slope includes zero. The conclusion regarding weak treatment linkage should specify that treatment linkage data were limited to a single cancer center registry and may not represent national performance.

The abstract should make clear that this is an analysis of aggregated routine program data and that the findings are primarily descriptive and programmatic, rather than evidence of clinical effectiveness or mortality impact.

Please give a frank account of the strengths and weaknesses of the article

The article addresses an important public health issue and has the potential to provide valuable national-level evidence on breast cancer early detection efforts in Bangladesh. Its use of routine program data, attention to geographic variation, and attempt to evaluate pandemic-related disruption are meaningful strengths.

However, the current version requires major revision. The main weaknesses are insufficient clarity about the data structure, uncertainty regarding whether coverage reflects unique women or total examinations, low and potentially differential reporting completeness, problematic interpretation of partial 2025 data, incomplete description of the interrupted time-series model, and overinterpretation of treatment linkage based on a single cancer center registry. These issues do not necessarily invalidate the study, but they substantially limit the strength of the conclusions.

With a robust revision that clarifies the data structure, improves the presentation of monthly and annual data, addresses the partial-year nature of 2025, provides sensitivity analyses, and tempers the conclusions, the manuscript could become a useful contribution to the literature on breast cancer early detection and health system monitoring in Bangladesh.

Reviewer #5: Thank you for the opportunity to review this paper, which is novel as the first to report CBE in Bangladesh, and with such a large dataset and timeseries. It is good to see the interrupted timeseries methodology used for the COVID assessment, the sensitivity assessments using alternative population estimates, and the benchmark to IARC. The benefits and limitations are well outlined in the discussion.

Suggested revisions:

- The use of the term 'screening' throughout the manuscript should be reviewed, it appears appropriate only in a couple of places in the discussion. CBE is not screening (as the authors acknowledge) - it is symptomatic examination, and is recommended for LMICs as a pre-requisite to investment in early detection and future mammographic screening (as the authors acknowledge this is prohibitively expensive and requires substantive infrastructure). Where the term screening is used in relation to the CBE programme data "CBE examinations" or "receipt of CBE" "undergoing CBE" or similar should be used instead. This is correct in line 149 but not elsewhere (eg line 124, abstract etc).

- The WHO GCBI initiative, 3 pillar recommendations, and 2023 guidelines which includes CBE programmatic guidance in LMICs is mentioned late in the discussion (line 225) but would be good to bring up into the introduction (eg line 65 where WHO is mentioned) to better situate the CBE context for an international audience, and locate this work in the Pillar 1 context. The current line 225 GBCI discussion of Pillar 1 was slightly confusing as it talks about the 60% KPI (which is 60% breast cancers being stage 1 and 2, or early diagnosis) in the same context as referring to 3% coverage, these are different metrics and it would be good to clarify that.

- The Bangladesh CBE programme is mentioned in a couple of places, and the discussion refers to "substantial programme scale up" (line 194) however it would be very helpful for an international audience to more fully describe the Bangladesh CBE programme either prior to the methods, or in the study setting in the methods. Particularly what was the programmatuc investment relative to the WHO GBCI recommendations (ie population awareness, training of health professionals, cancer centres, diagnostic services etc). It would help the reader interpret the findings and discussion content.

- Line 57-58 re high income countries - likely that timely diagnosis (as well as access to treatments) is important.

- Line 90 I don't know what a 'upazila' health complex is if the authors could just explain that term

- The results do not seem to include a paragraph on completeness, nor is there a table with this information (although in the discussion line 240 there is reference to 31% national completeness). There is a comment in the results regarding a zero result if >60% completeness was included. If the authors could include content in the results about data completeness across the relevant variables (time, national vs facility/geography) that would be beneficial.

- Although the authors say that STROBE reporting is used (which is good to see), it would be good to reference these reporting guidelines in the manuscript text and also to include the STROBE reporting output as supplementary.

- The authors reference the 1% positivity as lower than RCTs, and later refer to the limitations of a single registry linkage reporting to make this assessment. It would be good to be clearer in the data sources that this is only one tertiary centre registry data, and to state more clearly in the discussion that this is likely to be an underestimate (if they believe this to be the case as implied).

- The very low radiology % prior to tertiary centre also seemed noteworthy (although depending on the CBE programme description this may be due to the location of radiology relative to diagnostics). Similarly the >75% untreated seemed very high, the CBE programme description may also help the international reader interpret this figure if most treatment (eg surgery) is not able to be done outside of a tertiary centre?

- There seem to be a lot of tables and figures, potentially some tables could be combined.

**********

what does this mean?). If published, this will include your full peer review and any attached files.

If you choose “no”, your identity will remain anonymous but your review may still be made public.

**Do you want your identity to be public for this peer review?**  For information about this choice, including consent withdrawal, please see our Privacy Policy

Reviewer #1: No

Reviewer #2: No

Reviewer #3: No

Reviewer #4: No

Reviewer #5: No

**********

[NOTE: If reviewer comments were submitted as an attachment file, they will be attached to this email and accessible via the submission site. Please log into your account, locate the manuscript record, and check for the action link "View Attachments". If this link does not appear, there are no attachment files.]

To ensure your figures meet our technical requirements, please review our figure guidelines: https://journals.plos.org/plosone/s/figures

You may also use PLOS’s free figure tool, NAAS, to help you prepare publication quality figures: https://journals.plos.org/plosone/s/figures#loc-tools-for-figure-preparation.

NAAS will assess whether your figures meet our technical requirements by comparing each figure against our figure specifications.

# Author comment: Author response to Decision Letter 1

**DOI:** 10.1371/journal.pone.0361910.r002

8 Sep 2026

Thank you to the Academic Editor and reviewers for their careful and constructive comments. We have revised the manuscript extensively in response to all points raised. A detailed point-by-point response to the Academic Editor and all reviewers has been uploaded as a separate “Response to Reviewers” file, together with the clean revised manuscript and the marked-up manuscript with tracked changes.

Attachment Submitted filename: PONE-D-26-23766_Response_to_Reviewers.docx

# Peer review: Decision Letter 1

**DOI:** 10.1371/journal.pone.0361910.r003

**Authors:** Qi Zhou

13 Oct 2026

Dear Dr. Das,

Please submit your revised manuscript by Nov 12 2026 11:59PM. If you will need more time than this to complete your revisions, please reply to this message or contact the journal office at plosone@plos.org. When you're ready to submit your revision, log on to https://www.editorialmanager.com/pone/ and select the 'Submissions Needing Revision' folder to locate your manuscript file.

- A letter that responds to each point raised by the academic editor and reviewer(s). You should upload this letter as a separate file labeled 'Response to Reviewers'.
- A marked-up copy of your manuscript that highlights changes made to the original version. You should upload this as a separate file labeled 'Revised Manuscript with Track Changes'.
- An unmarked version of your revised paper without tracked changes. You should upload this as a separate file labeled 'Manuscript'.

If you would like to make changes to your financial disclosure, please include your updated statement in your cover letter. Guidelines for resubmitting your figure files are available below the reviewer comments at the end of this letter.

If applicable, we recommend that you deposit your laboratory protocols in protocols.io to enhance the reproducibility of your results. Protocols.io assigns your protocol its own identifier (DOI) so that it can be cited independently in the future. For instructions see: https://journals.plos.org/plosone/s/submission-guidelines#loc-laboratory-protocols. Additionally, PLOS One offers an option for publishing peer-reviewed Lab Protocol articles, which describe protocols hosted on protocols.io. Read more information on sharing protocols at https://plos.org/protocols?utm_medium=editorial-email&utm_source=authorletters&utm_campaign=protocols.

As the corresponding author, your ORCID iD is verified in the submission system and will appear in the published article. PLOS supports the use of ORCID, and we encourage all coauthors to register for an ORCID iD and use it as well. Please encourage your coauthors to verify their ORCID iD within the submission system before final acceptance, as unverified ORCID iDs will not appear in the published article. *Only*  the individual author can complete the verification step; PLOS staff *cannot*  verify ORCID iDs on behalf of authors.

We look forward to receiving your revised manuscript.

Kind regards,

Qi Zhou

Guest Editor

PLOS One

Journal Requirements:

1. If the reviewer comments include a recommendation to cite specific previously published works, please review and evaluate these publications to determine whether they are relevant and should be cited. There is no requirement to cite these works unless the editor has indicated otherwise.

2. Please review your reference list to ensure that it is complete and correct. If you have cited papers that have been retracted, please include the rationale for doing so in the manuscript text, or remove these references and replace them with relevant current references. Any changes to the reference list should be mentioned in the rebuttal letter that accompanies your revised manuscript. If you need to cite a retracted article, indicate the article’s retracted status in the References list and also include a citation and full reference for the retraction notice.

Additional Editor Comments:

The reviewers acknowledge the substantial improvements made to the manuscript and recognize the value of this large, country-level dataset in addressing an important public health issue. However, several concerns remain, particularly regarding data accuracy, consistency, and the completeness of statistical reporting. The authors are requested to carefully address each reviewer comment point by point, verify the underlying data and analyses, correct any identified errors, and revise the manuscript and supplementary materials accordingly. The study has considerable potential to contribute valuable evidence to the field, and I encourage the authors to undertake these remaining revisions carefully to further strengthen the rigor, reliability, and clarity of the manuscript.

[Note: HTML markup is below. Please do not edit.]

Reviewer's Responses to Questions

**Comments to the Author**

Reviewer #1: All comments have been addressed

Reviewer #2: All comments have been addressed

Reviewer #3: (No Response)

Reviewer #4: All comments have been addressed

Reviewer #5: (No Response)

**********

2. Is the manuscript technically sound, and do the data support the conclusions?

Reviewer #1: Yes

Reviewer #2: Yes

Reviewer #3: (No Response)

Reviewer #4: Yes

Reviewer #5: Yes

**********

3. Has the statistical analysis been performed appropriately and rigorously?

Reviewer #1: Yes

Reviewer #2: Yes

Reviewer #3: (No Response)

Reviewer #4: Yes

Reviewer #5: Yes

**********

4. Have the authors made all data underlying the findings in their manuscript fully available?

The PLOS Data policy

Reviewer #1: Yes

Reviewer #2: Yes

Reviewer #3: (No Response)

Reviewer #4: Yes

Reviewer #5: Yes

**********

5. Is the manuscript presented in an intelligible fashion and written in standard English?

Reviewer #1: Yes

Reviewer #2: Yes

Reviewer #3: (No Response)

Reviewer #4: Yes

Reviewer #5: Yes

**********

Reviewer #1: The revised manuscript represents a rigorous, transparent, and appropriately cautious analysis of routine CBE program data. The authors have successfully addressed all major concerns from the previous review. The study makes a meaningful contribution to understanding the performance and limitations of breast cancer early detection activities in Bangladesh and provides a model for similar analyses in other resource-limited settings.

Reviewer #2: Dear Authors, thanks for having addressed all the comments previously reported. The revised version of the manuscript has now sufficient standard of clearness and readability.

Reviewer #3: Thank you for the careful revision and detailed responses. The clearer description of reported examination events, inclusion of the full 2025 calendar year, removal of the rural–urban comparison, and more cautious interpretation of positivity, NICRH data, and the interrupted time-series analysis address most of my previous concerns. I have a few remaining requests:

1. Please verify the district denominators. Table 3 assigns 609,718 eligible women to Cumilla and 1,454,114 to Cox’s Bazar in 2025. This appears inconsistent with the stated proportional allocation from the 2022 census, which records a substantially larger female population in Cumilla than in Cox’s Bazar. Please check how district census populations were matched to examination counts across all years and update the geographic rates, rankings, and supplementary data if needed.

2. Please reconcile the facility-month count. The manuscript reports 32,178 facility-month records, whereas the annual submitted-report counts in S1 Table A4 sum to 32,044, matching the monthly counts in S3. If these figures refer to different types of records, please explain the distinction.

3. Please add reporting counts for the fixed facility cohorts. S1 Table A5 shows declining examination counts after 2023. The number of submitted facility-month reports, or examinations per submitted report, for each cohort and year would help readers assess how much changing reporting frequency contributed to that decline.

4. Please complete the interrupted time-series results in the supplement. The manuscript mentions analyses using an April 2020 interruption and autoregressive errors, but their estimates are not shown in S1 Table A6. Please report these results or qualify the statement about their agreement with the primary analysis. Please also check the fitted series in S3, which gives a negative fitted examination count for April 2019.

5. Please clarify the description of the geographic codes. Although S2 omits district names, exact counts in Table 3 allow some codes to be matched to named districts. Please describe the data as coded rather than fully anonymized and ensure that this presentation meets the journal’s request concerning location data.

Reviewer #4: The authors have satisfactorily addressed my substantive comments from the previous round of review. In particular, the revised manuscript now appropriately characterizes the facility-month structure of the routine program data, incorporates complete 2025 data, substantially improves the interrupted time-series analysis and its interpretation, explicitly addresses incomplete and differential reporting, and appropriately limits the interpretation of CBE positivity and the contextual NICRH registry data.

The revised framing also better distinguishes reported program activity from population screening coverage and appropriately acknowledges the limitations arising from event-level rather than individual-level data, incomplete reporting, the short pre-intervention period, and the absence of individual diagnostic and treatment pathways.

I have no further substantive methodological concerns arising from my previous comments.

Reviewer #5: Thank you, the authors have made substantial changes to the paper including fully revised analyses, appropriate reframing and improved descriptors of the data, country level context, cautions in interpretation and recommendations. It retains value as a very large dataset at a country level on an important topic.

The manuscript does not need to reflect the revised analyses suggest removing from the text (or briefly revising content):

Line 475

For these reasons, our previous characterization of facility-level differences as rural-versus-urban population inequity was not supported by the available data and has been replaced by a descriptive analysis of facility type.

And Line 538

The revised analysis includes the complete 2025 calendar year, explicit measurement of reporting completeness, facility-type analyses, denominator and high-volume-facility sensitivity analyses, fixed cohorts of consistently reporting facilities, and a seasonally adjusted interrupted time-series model with autocorrelation-robust inference.

Very minor

Line 351

Reporting completeness remained incomplete throughout the study period

Duplicate wording, could remove the word completeness: Reporting remained incomplete throughout the study period.

**********

what does this mean?). If published, this will include your full peer review and any attached files.

If you choose “no”, your identity will remain anonymous but your review may still be made public.

**Do you want your identity to be public for this peer review?**  For information about this choice, including consent withdrawal, please see our Privacy Policy

Reviewer #1: No

Reviewer #2: No

Reviewer #3: No

Reviewer #4: No

Reviewer #5: No

**********

[NOTE: If reviewer comments were submitted as an attachment file, they will be attached to this email and accessible via the submission site. Please log into your account, locate the manuscript record, and check for the action link "View Attachments". If this link does not appear, there are no attachment files.]

To ensure your figures meet our technical requirements, please review our figure guidelines: https://journals.plos.org/plosone/s/figures

You may also use PLOS’s free figure tool, NAAS, to help you prepare publication quality figures: https://journals.plos.org/plosone/s/figures#loc-tools-for-figure-preparation.

NAAS will assess whether your figures meet our technical requirements by comparing each figure against our figure specifications.

# Author comment: Author response to Decision Letter 2

**DOI:** 10.1371/journal.pone.0361910.r004

13 Oct 2026

We thank the Academic Editor and reviewers for their careful evaluation of our manuscript and their constructive comments. We have addressed the points raised in this second revision. A detailed, point-by-point response to all editorial and reviewer comments has been provided in the separately uploaded Response to Reviewers document. The corresponding revisions are reflected in the clean and tracked-changes versions of the manuscript.

Attachment Submitted filename: PONE-D-26-23766_R2_Response_to_Reviewers.docx

# Editor report: Decision Letter 2

**DOI:** 10.1371/journal.pone.0361910.r005

**Authors:** Qi Zhou

19 Oct 2026

Reported clinical breast examination activity in Bangladesh, 2019–2025: temporal trends, geographic variation, positivity, and reporting completeness

PONE-D-26-23766R2

Dear Dr. Das,

We’re pleased to inform you that your manuscript has been judged scientifically suitable for publication and will be formally accepted for publication once it meets all outstanding technical requirements.

Within one week, you’ll receive an e-mail detailing the required amendments. When these have been addressed, you’ll receive a formal acceptance letter and your manuscript will be scheduled for publication.

An invoice will be generated when your article is formally accepted. Please note, if your institution has a publishing partnership with PLOS and your article meets the relevant criteria, all or part of your publication costs will be covered. Please make sure your user information is up-to-date by logging into Editorial Manager at Editorial Manager® and clicking the ‘Update My Information' link at the top of the page. For questions related to billing, please contact billing support.

If your institution or institutions have a press office, please notify them about your upcoming paper to help maximize its impact. If they’ll be preparing press materials, please inform our press team as soon as possible -- no later than 48 hours after receiving the formal acceptance. Your manuscript will remain under strict press embargo until 2 pm Eastern Time on the date of publication. For more information, please contact onepress@plos.org.

Kind regards,

Qi Zhou

Guest Editor

PLOS One

Additional Editor Comments (optional):

Reviewers' comments:

# Editor report: Acceptance letter

**DOI:** 10.1371/journal.pone.0361910.r006

**Authors:** Qi Zhou

PONE-D-26-23766R2

PLOS One

Dear Dr. Das,

I'm pleased to inform you that your manuscript has been deemed suitable for publication in PLOS One. Congratulations! Your manuscript is now being handed over to our production team.

At this stage, our production department will prepare your paper for publication. This includes ensuring the following:

* All references, tables, and figures are properly cited

* All relevant supporting information is included in the manuscript submission,

* There are no issues that prevent the paper from being properly typeset

You will receive further instructions from the production team, including instructions on how to review your proof when it is ready. Please keep in mind that we are working through a large volume of accepted articles, so please give us a few days to review your paper and let you know the next and final steps.

Lastly, if your institution or institutions have a press office, please let them know about your upcoming paper now to help maximize its impact. If they'll be preparing press materials, please inform our press team within the next 48 hours. Your manuscript will remain under strict press embargo until 2 pm Eastern Time on the date of publication. For more information, please contact onepress@plos.org.

You will receive an invoice from PLOS for your publication fee after your manuscript has reached the completed accept phase. If you receive an email requesting payment before acceptance or for any other service, this may be a phishing scheme. Learn how to identify phishing emails and protect your accounts at https://explore.plos.org/phishing.

If we can help with anything else, please email us at customercare@plos.org.

Thank you for submitting your work to PLOS One and supporting open access.

Kind regards,

PLOS One Editorial Office Staff

on behalf of

Dr. Qi Zhou

Guest Editor

PLOS One
